# IFIT2-induced transcriptomic changes in *Mycobacterium tuberculosis* infected macrophages

**DOI:** 10.3389/fcimb.2025.1536446

**Published:** 2025-05-20

**Authors:** Ewura-Esi Manful, Ray-Dean Pietersen, Bienyameen Baker

**Affiliations:** DSI-NRF Centre of Excellence for Biomedical Tuberculosis Research; South African Medical Research Council Centre for Tuberculosis Research; Division of Molecular Biology and Human Genetics, Faculty of Medicine and Health Sciences, Stellenbosch University, Cape Town, South Africa

**Keywords:** IFIT2, macrophages, tuberculosis, transcriptomics, gene expression, host-pathogen interaction

## Abstract

Interferon-induced protein with tetratricopeptide repeat 2 (IFIT2) is known for its antiviral activity and has recently been implicated in the defense against *Mycobacterium tuberculosis (M. tb).* This study employed global transcriptomics to investigate the downstream effects of IFIT2 induction in THP-1 macrophages infected with R179 *M. tb*, aiming to elucidate its role and downstream contributing factors that aid it in intracellular *M. tb* killing. Using a vector-based overexpression approach, IFIT2 expression was induced in THP-1 cells infected with R179 *M. tb*, followed by RNA extraction 12 hours post-infection and AmpliSeq-based targeted transcriptome sequencing. Bioinformatics analysis identified 282 differentially expressed genes (DEGs), of which 189 were upregulated and 90 were downregulated (FDR <0.05). Filtering for highly significant DEGs (|log_2_(fold change) | > 1.5) yielded 70 genes, predominantly upregulated, with functional enrichment in pathways such as defense response to viruses and cytokine-mediated signaling. Signaling pathway impact analysis highlighted pathway activation and inhibition of the tuberculosis (TB) pathway. RT-qPCR validation confirmed the upregulation of selected DEGs (ISG15, CMPK2, RSAD2, IFI44L, IFI44), corroborating the AmpliSeq data. This study provides comprehensive insights into the transcriptomic profile induced by IFIT2 in TB, revealing critical downstream contributors and pathways that underpin IFIT2’s ability to combat *M. tb* infection.

## Introduction

Tuberculosis (TB), caused by *Mycobacterium tuberculosis* (*M. tb*) is a global health issue that claims millions of lives every year. According to estimates from WHO, 10.8 million people worldwide developed TB in 2023 with 1.25 million deaths which is a slight increase from 10.7 million cases of TB and a decline from 1.3 million deaths in 2022 (WHO, 2024) Despite several interventions made in the treatment strategies for TB, the emergence of drug-resistant strains is on the rise with multidrug-resistant (MDR) and extensively drug-resistant (XDR) strains becoming most common, suggesting the need for new therapeutic approaches. Therefore, targeting host genes as potential host-directed therapies (HDTs) are currently gaining traction as a rational approach for improved TB therapy because of the unlikelihood of host resistance development (Palucci and Delogu, 2018). HDTs are a class of therapeutic agents that target the host immune system by improving their ability to fight infections rather than directly attacking the pathogen causing the disease (Palucci and Delogu, 2018).

During the host immune response to viruses, interferons (IFN) coordinate a strong host immune response by activating over 300 interferon-stimulated genes (ISGs) (Schoggins, 2018). These ISGs encode a variety of proteins that impede several phases of the viral replication cycle, including entry into the host cells, protein translation, replication, virus particle assembly, and propagation (Diamond and Farzan, 2013). The transcription of hundreds of ISGs downstream of IFN signaling has also been studied as an important factor for efficient host defense (Schoggins, 2018). IFNs are known to influence the host immune response to *M. tb* infection (Donovan et al., 2017), in addition to eliciting antiviral responses. They have been widely used in combination with antimicrobial drugs to treat patients with TB and have been shown to have a wide range of effects on *M. tb* infection. For example, Type I IFNs have been shown to enhance *M. tb* infection in the vast majority of cases, whereas IFN-γ (gamma) has been shown to protect the host against *M. tb* infection (Travar et al., 2016). Among the most potently induced ISGs are the interferon-induced protein with tetratricopeptide repeats (IFITs).

IFIT family members are known for their antiviral activity. They are known to prevent viral replication by binding to and controlling viral proteins and RNAs (Mears and Sweeney, 2018). IFIT genes code for a group of proteins that are activated by IFN, viral infection, or pathogen-associated molecular pattern (PAMP) recognition. They have molecular weights ranging between 47 and 72kDa and they are conserved in all vertebrates that have coevolved with the IFN system during the evolution of the adaptive immune system. In humans, four members of the IFITs family have been identified: IFIT1 (ISG56), IFIT2 (ISG54), IFIT3 (ISG60), and IFIT5 (ISG58), all of which are found on chromosome 10q23 (Mears and Sweeney, 2018). Despite their roles in the defense against viruses, their roles and relationships in macrophages and other immune cells involved in the fight against *M. tb* have not yet been established. However, a previous study on the transcriptome profile of human monocyte-derived macrophages infected with both pathogenic and non-pathogenic mycobacteria identified the IFITs protein family as the top upregulated genes involved in the killing of mycobacteria (Madhvi et al., 2022). Additionally, the expression levels of IFIT genes were found to be higher in individuals with latent TB infection than in those with active TB, suggesting their potential role in controlling TB infection (Madhvi et al., 2022). To explore the involvement of IFIT genes in TB infection, their expression was stimulated to assess their impact on mycobacterial growth. The results showed that overexpression (knock-up) of IFITs led to a decrease in mycobacterial growth by approximately 48%, whereas gene knockdown resulted in a 78% increase in mycobacterial growth (Madhvi et al., 2022).

Emerging research has highlighted the involvement of IFIT genes in cancer growth and metastasis, revealing their opposing oncogenic functions. This is a crucial consideration when evaluating IFITs as potential therapeutic targets for TB. Specifically, IFIT1, IFIT3, and IFIT5 have been shown to enhance cancer cell growth (Pidugu et al., 2019), making them less desirable for further investigation regarding their roles in mycobacterial killing. Conversely, IFIT2 has been identified as a tumor suppressor gene and numerous studies have demonstrated that its knockdown significantly increases cancer cell proliferation in various tumors (Pidugu et al., 2019). This suggests a potentially positive prognosis for patients with cancer undergoing TB treatment that targets IFIT2. Moreover, a link between TB and cancer has been implicated in several studies (Yu et al., 2008; Wu et al., 2011; Heuvers et al., 2012), further underscoring the importance of carefully selecting IFIT genes for TB therapeutic development.

Considering the well-documented antiviral functions of IFIT2, its role in TB infection and mechanisms of action remain poorly understood. Understanding the downstream effects of IFIT2 expression in macrophages could provide new insights into host defense mechanisms against *M. tb* and identify potential targets for therapeutic interventions. Therefore, our study aimed to elucidate the downstream contributors to IFIT2’s antimycobacterial effects in macrophages infected with mycobacteria. Using global transcriptomics, our findings revealed key differentially expressed genes (DEGs) upregulated downstream of IFIT2 induction. The increased expression of chemokines, cytokines, and other interferon-stimulated genes (ISGs) demonstrated antimycobacterial killing effects and involvement in mycobacterial infections. These diverse gene expression profiles were associated with Gene Ontology (GO) and Kyoto Encyclopedia of Genes and Genomes (KEGG) pathways, including responses to viruses, innate immune responses, and chemokine signaling pathways. Additionally, we observed from our Signaling Impact analysis that the tuberculosis pathway was inhibited confirming the downstream contributing factors to the antimycobacterial activity of IFIT2.

The insights gained from this study will advance our understanding of the molecular mechanisms by which IFIT2 contributes to the intracellular killing of *M. tb*. By delineating the transcriptomic profile associated with IFIT2 induction in TB, we aimed to identify potential therapeutic targets and pathways that could be leveraged to improve TB treatment strategies. Ultimately, this study seeks to contribute to the development of more effective interventions for TB, addressing the critical need for global health.

## Methods

### Macrophage and mycobacteria cell culture

THP-1 cells, which are a human monocytic cell line, were cultured in a T25 cell culture flask in Roswell Park Memorial Institute Medium (RPMI)-1640 (LTC Tech, 21875034) supplemented with 10% heat-inactivated fetal bovine serum (FBS) (LTC Tech, 10493106), incubated under 5% CO_2_ and at 37°C and transferred after 3 days to a T75 culture flask for further expansion inside a biosafety level 2 laboratory (BSL2).

The clinical multi drug-resistant (MDR) R179 *M. tb* strain and the *M. Bovis* BCG strain were cultured separately in Middlebrook 7H9 (Sigma-Aldrich, M0178) with 10% OADC (Albumin, Dextrose, Oleic Acid, Catalase and Sodium Chloride) (Becton Dickinson, 212240) and 0.5% glycerol (Merck Millipore, Germany, G5516-1L) without Tween 80, as this detergent is known to affect macrophage uptake and the host response to *M. tb* (Leisching et al., 2016b),. The bacteria were cultured as previously described. Briefly, a starter culture was set up in T25 cell culture flask at a volume of 10 ml and incubated for 2-3 days at 37°C until an optical density (OD) of less than 0.4 was reached. Subcultures, also in T25 cell culture flasks, were grown to a maximum OD of 0.4 and stored at -80°C until needed.

### Macrophage infection with mycobacteria

For infection experiments, THP-1 cells were seeded in 24-well plates (CELLSTAR^®^, P1PLA044C-000048) at 1 × 10^5^ cells/well with Phorbol 12-Myristate 13-Acetate (PMA) (Sigma Aldrich, USA) at a final concentration of 100 nM in growth medium (RPMI) supplemented with 10% FBS and incubated for 72 h to differentiate THP-1 cells into macrophage cells. The differentiated THP-1 (dTHP-1) cells were then transferred to a CO_2_ incubator in a biosafety level 3 (BSL3) laboratory and infected.

Mycobacterial culture stocks (1ml) were thawed, and clumps were broken and loosened by pipetting ten times with a 1000 μl pipette. Subsequently, the mycobacterial culture was syringed 10 times through a 25 gauge (G25) needle to further break the clumps and obtain single bacteria (Leisching et al., 2016a). This technique is referred to as the syringe-settle-filtrate (SSF) method (Madhvi et al., 2020). The vial was immediately allowed to stand for 30 s to allow the large clumps to settle after syringing. The top 750 μl was carefully removed, mixed with 4.25 ml macrophage growth medium and the 5 ml bacterial suspension filtered through a 5.0 μm pore size filter (Merck Millipore, Germany). Filtering through a 5.0 μm pore size filter eliminates all large clumps, leaving only small clumps of approximately 2–3 bacteria. More than 90% of the filtered bacteria have been determined to be single (Leisching et al., 2016a). The exact same method (SSF) was used prior to infection to determine the titer of the filtered bacteria. The required volume of filtered bacteria was mixed with growth medium and added to the dTHP-1 cells to give a Multiplicity of Infection (MOI) of one. The 24-well plates were incubated for 4 h to allow for bacterial uptake.

To remove extracellular mycobacteria, the infected dTHP-1 cells were washed three times with 800 μl 1x Phosphate Buffer saline (PBS) (LTC Tech, 14190144) and prepared for vector-based overexpression of the target gene (IFIT2) by adding 250 μl of growth medium per well. Cells that served as a negative control for vector treatment, received 300 μl of growth medium per well. Uninfected THP-1 cells were used as controls. For basal measurements, infected and uninfected cells were collected 4 h post-infection to capture baseline gene expression prior to vector-based treatments.

### Transfection of THP-1 for vector-based overexpression of target gene (IFIT2)

Plasmid transfection was performed using Mission siRNA liposome-based transfection reagent (Sigma-Aldrich, S1452). The transfection of the IFIT2 gene into THP-1 cells for its overexpression (knock-up) was done in 24 well plates with 1 × 10^5^ cells per well in 250 μl of growth medium. For each well containing THP-1 cells (infected or uninfected) that had to be treated with vector, 50 μl of transfection Master Mix was added to the 250 μl of growth medium already present in the wells. Transfection Master Mix, per well, consisted of 1 μl of transfection reagent, 0.3 μl (equating to 60 ng at the final dilution) of plasmid DNA vector and 48.7 μl of Dulbecco’s Modified Eagle’s Medium (DMEM) which was mixed, vortexed and allowed to stand at room temperature for 15 minutes. The final volume in each well was 300 μl.

The plasmid DNA vectors used included IFIT2 with a FLAG tag placed at the N-terminus [GenScript, SC1200_OHu31069C], IFIT2 with a FLAG tag placed at the C-terminus (GenScript, SC1200_OHu31069D), IFIT2 with no FLAG tag (GenScript, SC1200_OHu31069E), a non-specific gene vector, Creatinine Kinase (CKB) (GenScript, OHu09573), and Empty Vector (GenScript, SC1822). The pcDNA 3.1+C/-(K)-DYK plasmid was used to generate each vector. Uninfected THP-1 cells, mycobacteria-infected THP-1 cells, uninfected-IFIT2 induced THP-1 cells, mycobacteria-infected CKB-induced THP-1 cells, and mycobacteria-infected THP-1 cells transfected with the empty vector served as control samples. The cells were then incubated for 8 h at 37°C in an incubator with 5% CO_2_.

### RNA extraction

Infected THP-1 cells after 4 h of uptake (4 h post infection) and uninfected THP-1 cells were prepared for RNA extraction after washing thrice with PBS. RNA was extracted immediately after collecting the 4-hour uptake samples to accurately capture baseline gene expression. Total RNA from human macrophage-like cells was extracted using a kit (RNeasy Plus Mini Kit) according to the manufacturer’s instructions. Extraction was also performed immediately following the 12 h infection period. Genomic DNA (gDNA) was removed by column filtration using the “gDNA eliminator” column included in the kit. For each experiment, the RNA quantity and quality were assessed using an Agilent 2100 Bioanalyzer. Only RNA samples with an RNA integrity number (RIN) ≥ 9 were used for AmpliSeq (Targeted Transcriptome Sequencing) and RT-qPCR experiments. Total RNA was extracted and frozen immediately at −80°C until use.

### AmpliSeq (targeted transcriptome sequencing)

AmpliSeq, a targeted transcriptome sequencing technique, was performed at the Central Analytical Facilities (CAF) at Stellenbosch University, South Africa. The Ion AmpliSeq™ Transcriptome Human Gene Expression Panel Chef-Ready Kit was used to prepare Ion AmpliSeq transcriptome libraries on the Ion Chef™ System according to the manufacturer’s protocol. 10 ng total RNA was reverse transcribed using the SuperScript™ VILO™ cDNA Synthesis Kit with incubation at 42°C for 30 minutes and enzymatic inactivation at 85°C for 5 minutes on a SimpliAmp™ Thermal Cycler (Thermo Fisher Scientific). The reverse-transcribed samples were loaded onto the Ion Chef™ System for library construction, with thirteen amplification cycles consisting of 16 minutes of annealing and extension for amplification of the primer targets. The libraries were diluted to a target concentration of 60 pM (picomolar) for template preparation using the Ion 540 Chef Kit (Thermo Fisher Scientific). 25 μl of diluted, pooled barcoded library were loaded onto the Ion Chef liquid handler for template preparation and enrichment using Ion 540™ Chef Reagents, Solutions and Supplies according to the protocol, MAN0010851 F.0. The enriched spherical ion particles were loaded onto an Ion 540 chip. Massively parallel sequencing was performed on the Ion Torrent™ GeneStudio™ S5 Prime System using Sequencing Solutions and Reagents, according to the protocol MAN0010851 F.0. Flow space calibration and base-caller analysis were performed using standard analysis parameters in Torrent Suite Version 5.18.1 Software. The reads were quality trimmed during the base-calling step using a moving window of 30 base pairs and a cutoff quality value (QV) of 15. Mapping was performed using the Torrent Mapping Alignment Program (TMAP) included in Torrent Suit software V5.18.1. Reads were mapped to the hg19_AmpliSeq_Transcriptome_v1.1 reference genome, and only reads with an alignment length of 17 or more were retained. Read counts were generated using the AmpliSeq RNA plugin.

### Identification of differentially expressed genes

The analysis of differentially expressed genes (DEGs) was performed in R version 4.3.1, using the DESeq2 R package, which uses a negative binomial distribution model (Love et al., 2014). The Benjamini-Hochberg method was used to correct for multiple testing. P-adjusted (or false discovery rate) values less than 0.05 and absolute log_2_(fold change) > 1.5 were used to screen out the highly significant DEGs.

### Functional analysis

The top 70 genes with log_2_(fold change) >1.5 extracted were selected and GO functional analysis and KEGG pathway enrichment analysis (P < 0.05) were performed using the clusterProfiler package (Tianzhi. et al., 2021) in R software.

Functional analysis was performed by first performing over-representation analysis using Gene Ontology (GO) terms to identify which GO terms were overrepresented or underrepresented in the DEGs. The GO terms were grouped into biological processes, molecular functions, and cellular components. The clusterProfiler R package was used for over-representation analysis by performing statistical analyses using hypergeometric testing (Tianzhi. et al., 2021). All genes tested for differential gene analysis were used as background genes. Genes with p-adjusted values less than 0.05, and log_2_(fold change) > 1.5 or < –1.5, were used as significant gene lists. Bar plots were used to visualize over-representative GO terms in the differentially expressed genes. Functional class scoring analysis was performed using gene set enrichment analysis (GSEA) and gene sets from the Kyoto Encyclopedia of Genes and Genomes (KEGG). GSEA evaluates whether gene sets for certain biological pathways are enriched in the larger absolute log_2_(fold changes) by performing analysis using the log_2_ (fold changes) generated by DEGs analysis from DESeq2 for each gene. The clusterProfiler R package and Pathview tools were used to perform gene set enrichment and pathway analyses in R Studio. Here, the coordinated differential expression of a group of genes that share functional similarities was tested instead of single genes. The NA values and duplicates were removed prior to the analysis. The Pathview R package was used to transform the KEGG pathway data from clusterProfiler into pathway images. Finally, pathway topology analysis was performed using the Signaling Pathway Impact Analysis (SPIA) tool. The SPIA R package in R was used to include all substantial information that was not obtained from the GO and GSEA analysis (Tarca et al., 2009).

### Construction of a protein–protein interaction network

To investigate the relationships between the highly significant DEGs extracted with log_2_(fold change) > 1.5, protein-protein interaction networks were constructed using the Search Tool for the Retrieval of Interacting Genes/Proteins (STRING) database version 12.0 with default parameters.

### RT-qPCR

To validate the transcriptomic data, we selected five key DEGs and performed RT-qPCR. Good quality RNA (RIN>9, 0.8µg) was converted to cDNA using the Quantitect^®^ Reverse Transcription Kit. To ensure the removal of genomic DNA, “gDNA wipe-out buffer” was used. RT-qPCR amplification was performed using a LightCycler 96 system (Roche, Germany). The LightCycler^®^ 480 SYBR Green I Master and QuantiTect^®^ primers were used.

Hs-GAPDH and Hs-UBC were selected as reference genes. The amplification process involved 45 cycles at 95°C for 10s followed, 60°C for 10s and finally 72°C for 10s. Gene expression fold-changes were computed for infected macrophages without treatment and infected macrophages with treatment using calibrated, normalized relative quantities using the equation N =N_0_×2^Cp^. All RT-qPCRs were performed on RNA extracted from three separate experiments, with three technical replicates for each set of experiments. All biological replicates, a positive control, and a non-reverse transcription control were run in triplicate (along with the calibrator) according to MIQE Guidelines (Bustin et al., 2009).

### Statistical analysis

Data generated from RT-qPCR were analyzed using Light Cycler 96 SW 1.1 software to obtain the relative mRNA expression. The relative mRNA expressions were further analyzed using One-Way ANOVA and Tukey’s test for multiple test corrections to generate p-values and graphs in GraphPad Prism V8.

GraphPad Prism V8 was used to analyze the CFU count using One-Way ANOVA and Tukey’s test for multiple test corrections to generate p-values and graphs.

## Results

### Gene optimization for knock-up experiments using different vector constructs

In constructing plasmid vectors for overexpression analysis, DNA sequences of target genes are often altered to include tags for purification and detection purposes. Commonly used mammalian expression vectors, such as pcDNA3.1, can incorporate tags such as FLAG, Poly histidine tag (His-tag) and the glutathione S-transferase (Booth et al., 2018). This study used the FLAG tag for IFIT2 vector construction, which is advantageous due to its small size, hydrophilicity, and ease of detection (Yadav et al., 2016). The positioning of tags can affect protein stability, activity, and structure, necessitating investigation to ensure functionality. Therefore, the current study evaluated the effects of FLAG tag positioning on IFIT2 expression and anti-mycobacterial activity against *M. tb* in THP-1 cells by comparing N-terminal FLAG, C-terminal FLAG, and no FLAG vector constructs. The anti-mycobacterial effects were evaluated by recording CFUs, and gene expression levels were measured using RT-qPCR.

### Effect of FLAG tag positioning on IFIT2 expression and anti-mycobacterial activity

The positioning of the FLAG tag significantly influenced IFIT2 expression levels and anti-mycobacterial activity. The C-Flagged IFIT2 vector exhibited the lowest gene expression and highest CFU counts against *M. bovis* BCG (**Supplementary Figures 1A, B**), indicating reduced anti-mycobacterial activity. In contrast, both N-Flagged and No-Flag IFIT2 vectors demonstrated higher expression levels and significantly lower CFU counts, suggesting improved anti-mycobacterial activity. Interestingly, the empty vector control also showed some anti-mycobacterial effects and IFIT2 expression, likely due to a non-specific immune response. However, the anti-mycobacterial activity of the empty vector-transfected control group was lower than that of the N-Flagged and No-Flag vectors though the observed difference were not statistically significant.

To explore this further, we tested the N-Flagged and No-Flag constructs using the multi-drug-resistant R179 *M. tb* strain, which is closer to real-life TB infections. A non-specific gene vector, Creatine Kinase (CKB), was included as a control, based on previous data showing unchanged expression in infected macrophages. The results showed that the N-Flagged IFIT2 vector had the lowest CFUs, and higher IFIT2 gene expression compared to the No-Flag vector (**Supplementary Figures 2A, B**). Interestingly, both the empty vector and CKB also showed some anti-mycobacterial activity and IFIT2 expression. Collectively, these results highlight the N-Flagged IFIT2 construct as the most effective and suitable for further high throughput studies, while the C-Flagged construct was deemed unsuitable due to poor antimycobacterial activity.

### General analysis of comparative transcriptome

**Figure 1:**
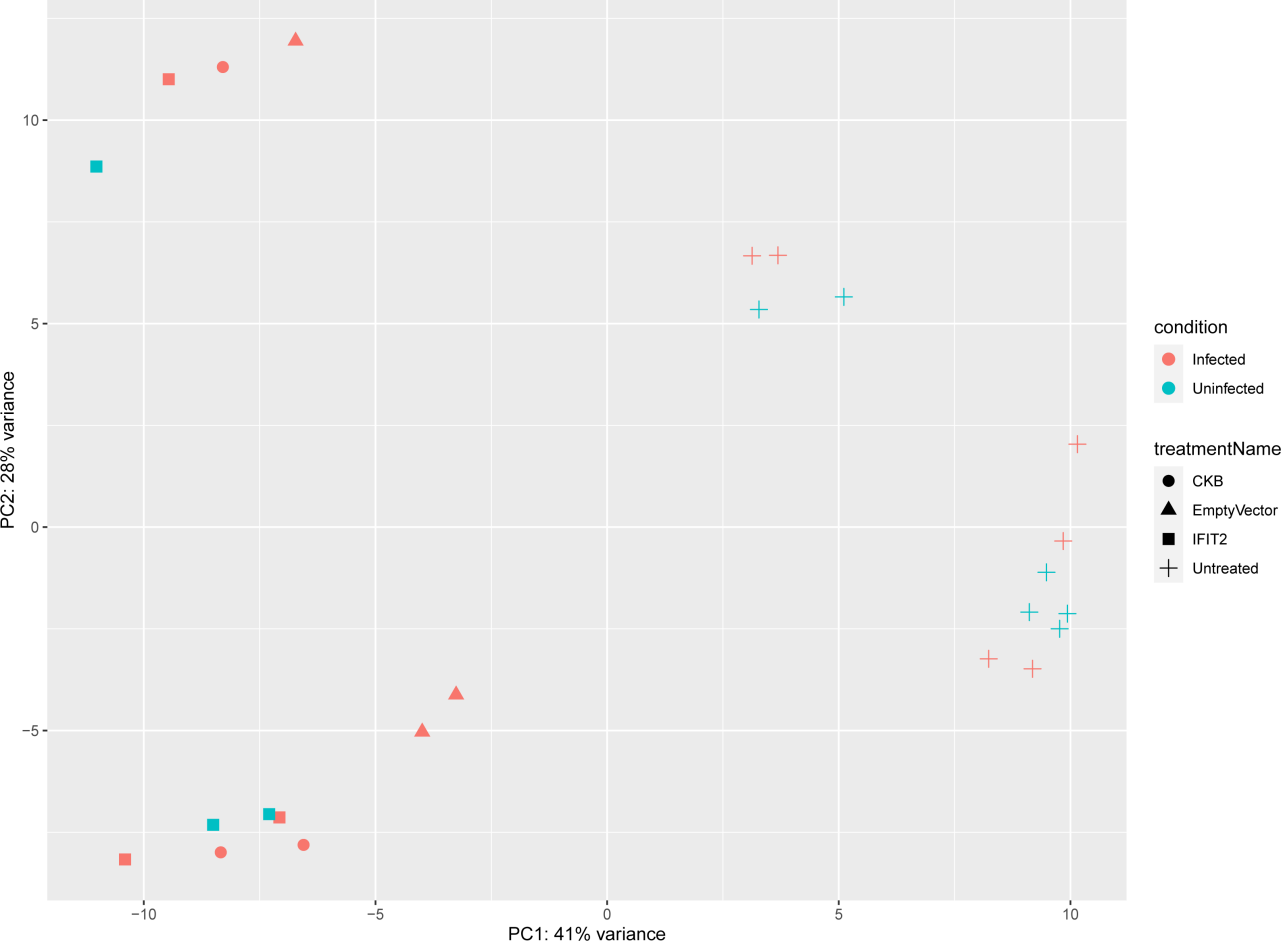
PCA plot illustrating sample grouping by condition and treatment. This plot shows how samples cluster based on their differences, with PC1 explaining 41% of the variance and PC2 explaining 28%. Samples are colored by condition (red for infected, blue for uninfected) and use different shapes to represent treatments (CKB, Empty Vector, IFIT2, and Untreated). The clustering clearly separates infected from uninfected samples and highlights differences between treatments, reflecting distinct patterns in the data.

RNA-seq analysis of the samples from uninfected cells, uninfected cells with IFIT2 treatment, infected cells with treatment (IFIT2, Empty vector, and non-specific gene vector CKB), and infected cells without treatment yielded an average of 63 million reads aligned to the hg19 AmpliSeq Transcriptome v1.1. Principal Component Analysis (PCA) showed distinct clustering of the different groups – uninfected from infected cells and treated from untreated cells (**Figure 1**). Infected, untreated THP-1 cells served as the reference for all treatments. The pairwise comparisons included IFIT2-transfected *M. tb*-infected THP-1 cells vs. *M. tb*-infected THP-1 cells only, empty vector-transfected *M. tb*-infected THP-1 cells vs. *M. tb*-infected THP-1 cells only, and CKB-transfected *M. tb*-infected THP-1 cells vs. *M. tb*-infected THP-1 cells only. Venn diagrams of the differentially expressed genes from the three treatment groups revealed both shared and unique genes in each treatment group (**Supplementary Figure 7**).

### Differential gene expression analysis and identification

Using R Studio and the DESeq2 R package, we conducted a thorough analysis to identify differentially expressed genes (DEGs) and compared all experimental treatments with the control groups. We employed a cutoff value of false discovery rate (FDR), also known as the p-adjusted value < 0.05, to extract significant DEGs. An MA-plot was generated to visualize genome-wide transcriptome expression at 12h post-infection in the treated groups compared to that in the control groups (**Supplementary Figure 3**). A total of 282 DEGs were identified, with 189 upregulated and 90 downregulated DEGs observed in IFIT2-induced *M. tb*-infected THP-1 cells compared to those in *M. tb*-infected THP-1 cells only. Due to the anti-mycobacterial activity and *IFIT2* gene expression levels observed in the control (empty vector-transfected *M. tb*-infected THP-1 cells, and the non-specific gene (*CKB*)-transfected *M. tb*-infected THP-1 cells) in the preliminary (gene optimization) experiments, we sought to identify the DEGs expressed (**Supplementary Figures 1**, **2**). For the Empty Vector, 194 DEGs were identified and compared to the *M. tb*-infected THP-1 cells only. Of these, 123 DEGs were upregulated and 71 DEGs were downregulated. In addition, 249 DEGs were identified for the non-specific gene vector CKB, of which 175 DEGs were upregulated and 74 DEGs were downregulated.

**Figure 2:**
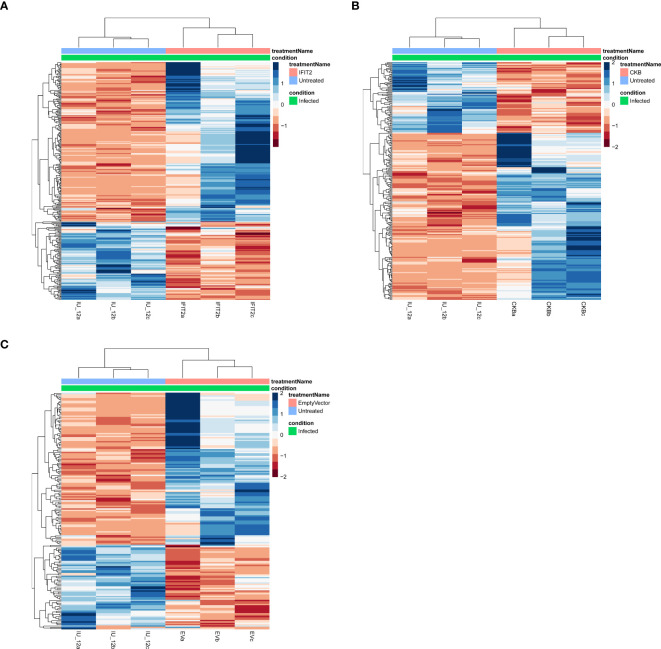
Heatmap for all treatments groups vs controls showing hierarchical clustering of differentially expressed genes (DEGs) in all samples. The infected untreated (IU) control groups were compared to the different treatment groups; **(A)** IFIT2-transfected THP-1 infected with R179M.tb, **(B)** CKB-transfected THP-1 infected with R179M.tb, and **(C)** Empty vector-transfected THP-1 infected with R179M.tb. Each column represents a sample with three biological replicates and each row represents a gene. The color scale represents the relative gene expression levels, with blue indicating high expression (upregulation), brown indicating low expression (downregulation), and white indicating no expression. DEGs were identified by false rate discovery (p-adjusted value) of < 0.05.

**Figure 3:**
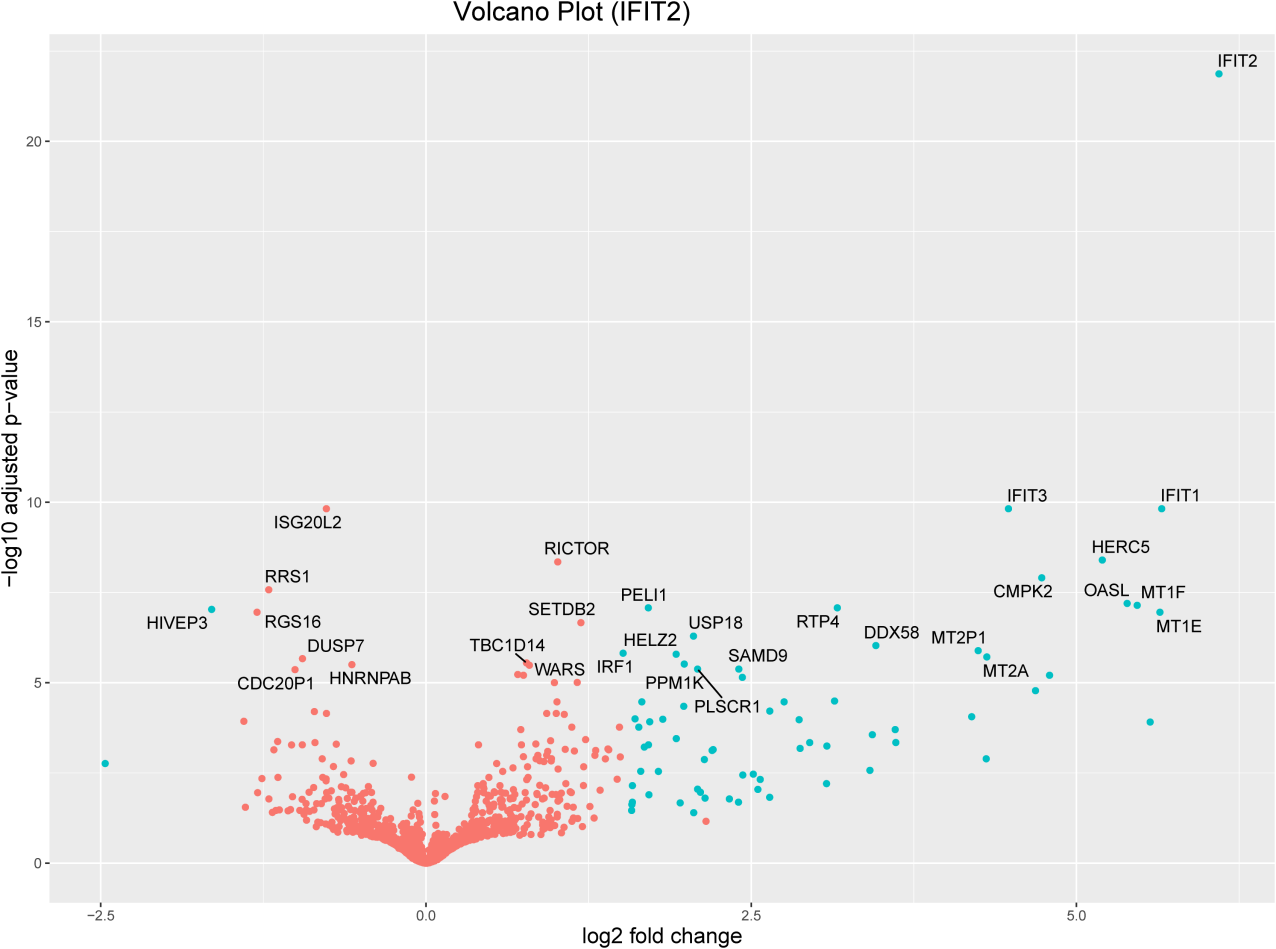
Volcano plot for the top 30 significant genes downstream of IFIT2 with an FDR<0.05. The blue dots represent genes with an absolute log_2_(fold change) greater than 1.5. Orange dots denote genes with an absolute log_2_(fold change) of less than 1.5. All dots above the zero point are upregulated genes and the ones below the zero point are downregulated genes.

**Figure 4:**
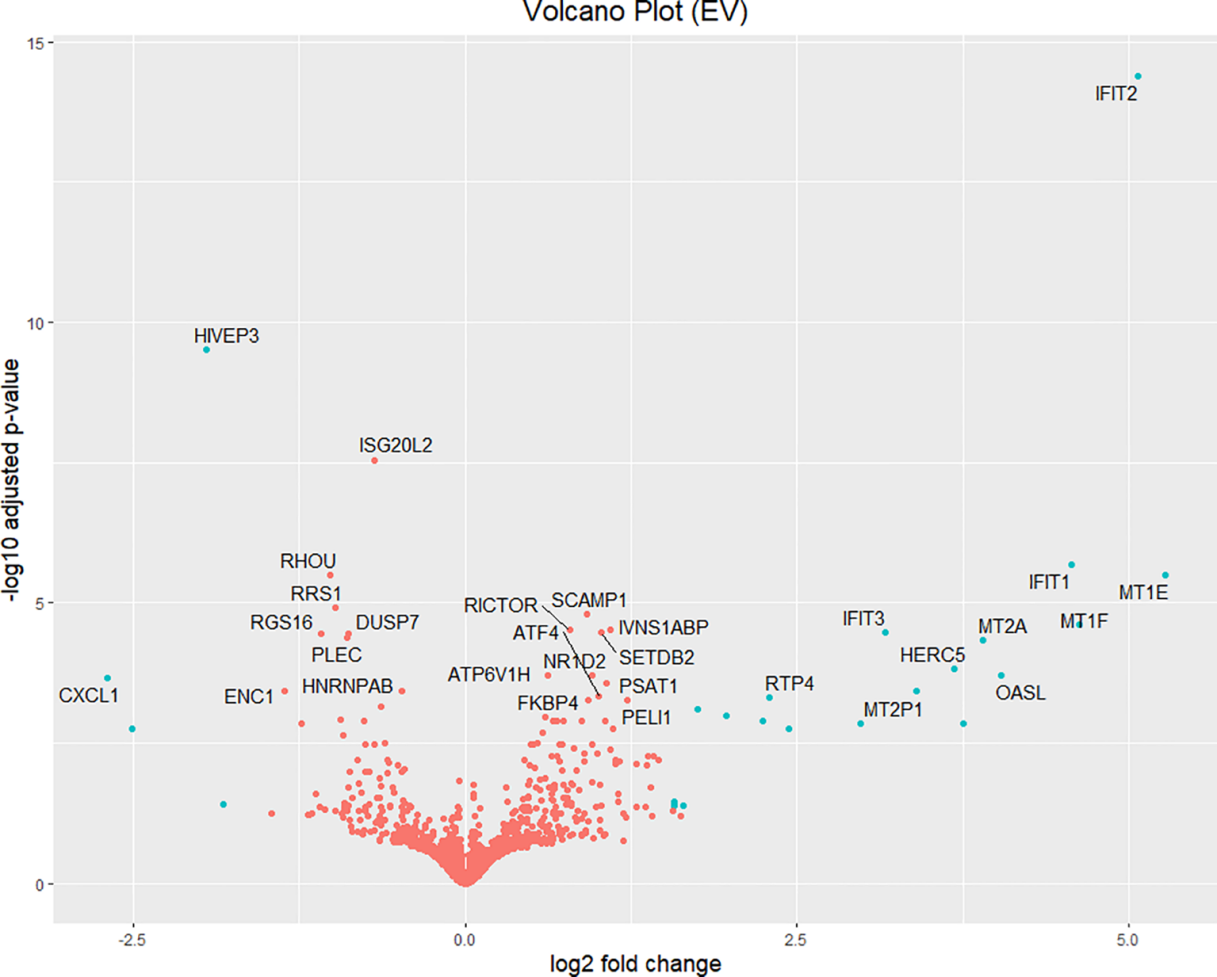
Volcano plot for Top 30 significant genes downstream of Empty vector with FDR<0.05. The blue dots represent genes with an absolute log_2_(fold-change) greater than 1.5. Orange dots denote genes with an absolute log_2_(fold change) of less than 1.5. All dots above the zero point are upregulated genes and the ones below the zero point are downregulated genes. EV: Empty Vector.

Volcano plots and heatmaps were used to provide detailed insights into the expression patterns of the DEGs. Hierarchical clustering of DEGs shown in the heatmap in **Figure 2**, revealed distinct clustering patterns between the treatment and control groups, underscoring the reliability of the samples. We used the gene expression data from each sample to determine the distance between them and their correlations. As shown in **Figure 2**, the treatment groups consistently formed separate clusters from those of the control groups, emphasizing the precision and reliability of the samples. Additionally, volcano plots in **Figures 3**, **4** illustrate the top 30 significant gene expression changes downstream of IFIT2 and the Empty Vector, respectively.

### Transcriptomic profile of *IFIT2*-induced *M. tb*-infected macrophages

We applied stringent filtering criteria to extract top-ranked significant DEGs, resulting in 70 (**Table 1**) genes with an absolute log_2_(fold change) > 1.5 and FDR < 0.05, among which only two were downregulated. The top 30 significant DEGs are represented in a volcano plot (**Figure 3**).

**Table 1:** All significantly expressed genes with p. adjusted value (FDR) < 0.05 & |log_2_(fold change)| > 1.5 downstream of *IFIT2* in *M. tb*-infected macrophages compared to control (infected macrophages only).

Gene	log_2_(fold change)	P value	P adjusted Value
*IFIT2*	6.095364	1.12E-26	1.35E-22
*IFIT1*	5.655365	5.00E-14	1.51E-10
*IFIT3*	4.477372	4.49E-14	1.51E-10
*HERC5*	5.198674	1.65E-12	4.00E-09
*CMPK2*	4.733145	7.17E-12	1.24E-08
*OASL*	5.389751	4.73E-11	6.36E-08
*MT1F*	5.466841	5.93E-11	7.18E-08
*PELI1*	1.709116	8.28E-11	8.43E-08
*RTP4*	3.161896	8.35E-11	8.43E-08
*HIVEP3*	-1.64719	1.00E-10	9.31E-08
*MT1E*	5.64155	1.38E-10	1.12E-07
*USP18*	2.056305	7.19E-10	5.12E-07
*DDX58*	3.458318	1.39E-09	9.35E-07
*MT2P1*	4.24462	2.03E-09	1.29E-06
*IRF1*	1.515355	2.52E-09	1.52E-06
*HELZ2*	1.922969	2.82E-09	1.62E-06
*MT2A*	4.311089	3.51E-09	1.93E-06
*PPM1K*	1.985364	6.29E-09	3.04E-06
*PLSCR1*	2.087734	1.00E-08	4.19E-06
*SAMD9*	2.404955	9.91E-09	4.19E-06
*MT1XP1*	4.793182	1.66E-08	6.22E-06
*SDS*	2.432066	2.00E-08	7.11E-06
*RSAD2*	4.685321	5.08E-08	1.66E-05
*TMEM140*	3.140736	1.01E-07	3.22E-05
*ATF4P3*	1.659223	1.15E-07	3.39E-05
*OAS1*	2.752892	1.13E-07	3.39E-05
*NADK*	1.981518	1.56E-07	4.51E-05
*IFIH1*	2.64276	2.17E-07	6.11E-05
*CXCL10*	4.194844	3.55E-07	8.76E-05
*APOL6*	1.607034	4.14E-07	0.0001
*PARP14*	1.820307	4.33E-07	0.000103
*EPSTI1*	2.868286	4.55E-07	0.000106
*FAM84B*	1.71979	5.40E-07	0.000121
*IFNB1*	5.566567	5.59E-07	0.000123
*DTX3L*	1.635955	8.17E-07	0.00017
*MX1*	3.606722	9.87E-07	0.000199
*GBP5*	3.431109	1.39E-06	0.000276
*PARP9*	1.924054	1.81E-06	0.000354
*MX2*	3.61166	2.48E-06	0.000454
*XAF1*	2.949697	2.56E-06	0.000456
*TRIB3*	1.710137	3.15E-06	0.000526
*TRIM22*	3.081727	3.57E-06	0.000569
*STC2*	1.678362	3.86E-06	0.000607
*CCL8*	2.875998	4.26E-06	0.000661
*BATF3*	2.207363	4.78E-06	0.000714
*IFI16*	2.199472	5.23E-06	0.000753
*CXCL11*	4.306407	1.02E-05	0.001283
*ISG15*	2.139982	1.09E-05	0.001345
*LZTS1*	-2.46576	1.50E-05	0.001733
*TNFSF13B*	3.412535	2.46E-05	0.002684
*ISG20*	1.650762	2.64E-05	0.002849
*NUPR1*	1.786633	2.68E-05	0.002871
*IFI44*	2.516045	3.25E-05	0.003417
*OAS2*	2.435235	3.49E-05	0.003607
*GBP4*	2.569444	4.99E-05	0.004791
*TNFSF10*	3.079018	6.76E-05	0.00625
*TAGAP*	1.587024	7.87E-05	0.007103
*SP110*	2.087771	0.000106	0.008901
*MB21D2*	2.551266	0.000108	0.009043
*C1orf115*	2.109667	0.000138	0.010842
*PROC*	1.713976	0.000185	0.012719
*IFI44L*	2.642011	0.000228	0.014978
*CD274*	2.145338	0.000245	0.015765
*IDO1*	2.33213	0.000266	0.016581
*RP11-44K6.4*	2.402228	0.000354	0.02041
*ARAP2*	1.586738	0.000359	0.020475
*SAMD9L*	1.954036	0.000376	0.021379
*FAS*	1.582688	0.000436	0.023752
*SLC30A1*	1.580979	0.000724	0.034599
*GBP1*	2.058111	0.000885	0.039979

### GO, KEGG and SPIA enrichment analysis for DEGs downstream of *IFIT2*

**Figure 5:**
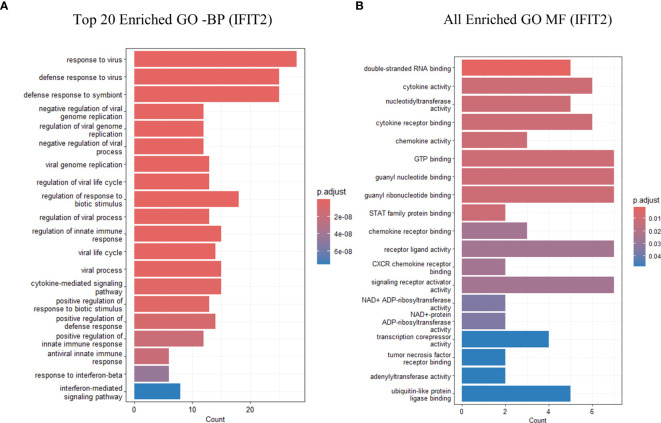
GO analysis showing only the top 20 significantly enriched pathways in *IFIT2* induced THP-1 cells with R179*M. tb*. **(A)** GO enriched terms for BP for IFIT2. **(B)** GO enriched terms for MF for IFIT2. The color of the bars indicates significance (adjusted P-value), and the size corresponds to the count of genes.

Gene Ontology (GO) enrichment analysis for biological processes highlighted enrichment in GO terms, including response to virus, defense response to virus, regulation of innate immune response, and cytokine-mediated signaling pathways. Molecular function analysis indicated enrichment in functions, such as double-stranded RNA binding, nucleotidyltransferase activity, GTP binding, and cytokine receptor binding. Notably, no GO terms were enriched for cellular components (**Figure 5**). A table showing GO terms with associated gene lists and statistical values can be found in **Tables 2**, **3**.

**Table 2:** Top 15 enrichment GO terms for Biological Process (BP) in THP-1 cells infected with R179M.tb after IFIT2 induction with their corresponding DEGs involved.

GOBP ID	Description	P. adjusted Value	Gene ID
GO:0009615	response to virus	2.6014E-27	*IFIT2/IFIT1/IFIT3/HERC5/OASL/RTP4/RIGI/IRF1/PLSCR1/RSAD2/OAS1/IFIH1/CXCL10/IFNB1/DTX3L/MX1/PARP9/MX2/TRIM22/CCL8/BATF3/IFI16/ISG15/ISG20/IFI44/OAS2/IFI44L/GBP1*
GO:0051607	defense response to virus	3.6276E-26	*IFIT2/IFIT1/IFIT3/HERC5/OASL/RTP4/RIGI/IRF1/PLSCR1/RSAD2/OAS1/IFIH1/CXCL10/IFNB1/DTX3L/MX1/PARP9/MX2/TRIM22/IFI16/ISG15/ISG20/OAS2/IFI44L/GBP1*
GO:0140546	defense response to symbiont	3.6276E-26	*IFIT2/IFIT1/IFIT3/HERC5/OASL/RTP4/RIGI/IRF1/PLSCR1/RSAD2/OAS1/IFIH1/CXCL10/IFNB1/DTX3L/MX1/PARP9/MX2/TRIM22/IFI16/ISG15/ISG20/OAS2/IFI44L/GBP1*
GO:0045071	negative regulation of viral genome replication	1.014E-16	*IFIT1/OASL/PLSCR1/RSAD2/OAS1/IFIH1/IFNB1/MX1/IFI16/ISG15/ISG20/OAS2*
GO:0045069	regulation of viral genome replication	2.2522E-14	*IFIT1/OASL/PLSCR1/RSAD2/OAS1/IFIH1/IFNB1/MX1/IFI16/ISG15/ISG20/OAS2*
GO:0048525	negative regulation of viral process	3.3516E-14	*IFIT1/OASL/PLSCR1/RSAD2/OAS1/IFIH1/IFNB1/MX1/IFI16/ISG15/ISG20/OAS2*
GO:0019079	viral genome replication	6.4748E-14	*IFIT1/OASL/PLSCR1/RSAD2/OAS1/IFIH1/IFNB1/MX1/CCL8/IFI16/ISG15/ISG20/OAS2*
GO:1903900	regulation of viral life cycle	2.2472E-13	*IFIT1/OASL/PLSCR1/RSAD2/OAS1/IFIH1/IFNB1/MX1/TRIM22/IFI16/ISG15/ISG20/OAS2*
GO:0002831	regulation of response to biotic stimulus	8.4823E-13	*HERC5/OASL/PELI1/USP18/RIGI/IRF1/PLSCR1/RSAD2/OAS1/IFIH1/PARP14/IFNB1/DTX3L/GBP5/PARP9/IFI16/ISG15/CD274*
GO:0050792	regulation of viral process	1.1955E-12	*IFIT1/OASL/PLSCR1/RSAD2/OAS1/IFIH1/IFNB1/MX1/TRIM22/IFI16/ISG15/ISG20/OAS2*
GO:0045088	regulation of innate immune response	1.0132E-10	*OASL/PELI1/USP18/RIGI/IRF1/PLSCR1/RSAD2/OAS1/IFIH1/PARP14/IFNB1/GBP5/PARP9/IFI16/ISG15*
GO:0019058	viral life cycle	2.6963E-10	*IFIT1/OASL/PLSCR1/RSAD2/OAS1/IFIH1/IFNB1/MX1/TRIM22/CCL8/IFI16/ISG15/ISG20/OAS2*
GO:0016032	viral process	7.272E-10	*IFIT1/OASL/PLSCR1/RSAD2/OAS1/IFIH1/IFNB1/MX1/PARP9/TRIM22/CCL8/IFI16/ISG15/ISG20/OAS2*
GO:0019221	cytokine-mediated signaling pathway	3.3289E-09	*OASL/USP18/IRF1/OAS1/CXCL10/PARP14/IFNB1/MX1/PARP9/CCL8/CXCL11/ISG15/TNFSF13B/OAS2/FAS*

**Table 3:** All GO terms for Molecular Function (GOMF) in THP-1 cells infected with R179*M.tb* after IFIT2 induction with their corresponding DEGs involved.

GOMF ID	Description	P. Adjusted Value	Gene ID
GO:0003725	double-stranded RNA binding	0.003781	*OASL/RIGI/OAS1/IFIH1/EIF2AK2/OAS2/SLC3A2/DHX58*
GO:0016779	nucleotidyltransferase activity	0.013512	*OAS1/PARP14/PARP9/PARP12/CGAS/OAS2/MB21D2/DKC1/PNPT1/POLR1F*
GO:0005525	GTP binding	0.039507	*RIGI/RHOU/RND3/MX1/GBP5/MX2/CGAS/GBP4/GTPBP1/TUBA1C/IFI44L/FKBP4/EHD1/RAB29/GBP1*
GO:0005126	cytokine receptor binding	0.039507	*CXCL10/IFNB1/CCL8/CXCL11/TNFSF13B/CXCL1/TNFSF10/STAT1/SMAD7/MYD88/TNF/IL1A*
GO:0005125	cytokine activity	0.039507	*CXCL10/IFNB1/CCL8/CXCL11/TNFSF13B/CXCL1/TNFSF10/TNF/SECTM1/BMP2/IL1A*
GO:0032813	tumor necrosis factor receptor superfamily binding	0.039507	*TNFSF13B/TNFSF10/STAT1/MYD88/TNF*
GO:0019001	guanyl nucleotide binding	0.039507	*RIGI/RHOU/RND3/MX1/GBP5/MX2/CGAS/GBP4/GTPBP1/TUBA1C/IFI44L/FKBP4/EHD1/RAB29/GBP1*
GO:0032561	guanyl ribonucleotide binding	0.039507	*RIGI/RHOU/RND3/MX1/GBP5/MX2/CGAS/GBP4/GTPBP1/TUBA1C/IFI44L/FKBP4/EHD1/RAB29/GBP1*
GO:0003712	transcription coregulator activity	0.039507	*HELZ2/PARP14/PARP9/TRIB3/TRIM22/TRIM25/TFAP2A/CBX4/NUPR1/TRIM5/TBL1XR1/TRIM21/HMGA2/JMY/AKIRIN2/PTPN14/IRF4*
GO:0005164	tumor necrosis factor receptor binding	0.039507	*TNFSF13B/TNFSF10/STAT1/TNF*
GO:0046332	SMAD binding	0.039507	*RGCC/HMGA2/PML/SMAD7/DAB2/BMP2*
GO:0019787	ubiquitin-like protein transferase activity	0.048224	*HERC5/PELI1/TRIM26/DTX3L/TRIM22/TRIM25/CBX4/TRIM5/MALT1/RNF13/UBE2C/TRIM21/HERC6/RNF149/PML/UBE2L6*

Additionally, Gene Set Enrichment Analysis (GSEA) using the KEGG database revealed enrichment in pathways related to viral infections, including influenza A, Hepatitis C, measles, and COVID-19, as well as pathways involved in host response to various pathogens, such as NOD-like receptor signaling and cytokine-cytokine receptor signaling pathways. The overlap of genes in these pathways was depicted using an Upsetplot, illustrating the shared genes among certain pathways (**Supplementary Figure 4**). A table showing specific KEGG pathways with associated gene lists, statistical values and enrichment score can be found in **Table 4**.

**Table 4:** All Enriched KEGG Pathways in DEGs in THP-1 cells infected with R179*M.tb* after *IFIT2* induction with their corresponding enrichment scores.

KEGG ID	Description	Enrichment Score
hsa05164	Influenza A	0.877667241
hsa05160	Hepatitis C	0.882222883
hsa05162	Measles	0.870922213
hsa05171	Coronavirus disease - COVID-19	0.836494333
hsa04061	Viral protein interaction with cytokine and cytokine receptor	0.875612928
hsa04621	NOD-like receptor signaling pathway	0.807225693
hsa04622	RIG-I-like receptor signaling pathway	0.89262838
hsa04060	Cytokine-cytokine receptor interaction	0.777227106
hsa04620	Toll-like receptor signaling pathway	0.855092635
hsa04623	Cytosolic DNA-sensing pathway	0.859308314
hsa05169	Epstein-Barr virus infection	0.770110534
hsa05165	Human papillomavirus infection	0.73215752

Signaling Pathway Impact Analysis (SPIA) further supported these findings, indicating significant enrichment and activation of pathways related to cytokine-cytokine receptor interaction and chemokine signaling. Evidence suggested inhibition of TB and activation of apoptosis pathways (**Supplementary Figure 5**; **Table 5**).

**Table 5:** Top 15 Pathways enriched for SPIA analysis after IFIT2 induction in THP-1 cells infected with R179*M.tb* and their corresponding statuses.

Pathway ID	Pathway	pPERT	pGFdr	Status
5164	Influenza A	0.13	1.15E-12	Activated
5162	Measles	0.015	4.47E-09	Activated
5168	Herpes simplex infection	0.003	2.26E-08	Activated
4060	Cytokine-cytokine receptor interaction	0.004	5.95E-07	Activated
5160	Hepatitis C	0.247	2.93E-06	Activated
4622	RIG-I-like receptor signaling pathway	0.139	4.44E-06	Activated
4620	Toll-like receptor signaling pathway	0.039	0.000137899	Activated
5152	Tuberculosis	0.736	0.002367588	Inhibited
4978	Mineral absorption	1	0.002374233	Inhibited
4062	Chemokine signaling pathway	0.043	0.005882018	Activated
4064	NF-kappa B signaling pathway	0.125	0.005882018	Activated
5202	Transcriptional misregulation in cancer	1	0.00704798	Inhibited
4623	Cytosolic DNA-sensing pathway	0.02	0.00704798	Activated
4210	Apoptosis	0.033	0.00704798	Activated
5143	African trypanosomiasis	0.322	0.009444797	Inhibited

### Empty vector: an inducer of non-specific immune response against *M. tb*

As seen from the preliminary experiment, the empty vector control revealed unexpected observations and outcome by showing an appreciable increase in IFIT2 gene expression and revealing antimycobacterial effects. We therefore hypothesized that the empty vector elicits a strong non-specific host-immune response in TB infection. As a result, we carried out a transcriptomic investigation to understand this outcome and tested out this hypothesis. It can be seen from **Table 6** that 23 DEGs were identified with 4 being downregulated and 19 upregulated after extracting genes with absolute log_2_(fold change) > 1.5. The top 30 significant genes with FDR <0.05 affected by empty is represented in a volcano plot (**Figure 4**).

**Table 6:** All significantly expressed genes with p. adjusted value < 0.05 & |log_2_(fold change)| > 1.5 in empty vector-transfected *M. tb*-infected macrophages compared to control (macrophage-infected cells only).

Gene	log_2_(fold change)	P value	P adjusted Value
*IFIT2*	5.068234	3.17E-19	3.97E-15
*HIVEP3*	-1.95131	4.95E-14	3.10E-10
*IFIT1*	4.566209	6.47E-10	2.03E-06
*MT1E*	5.270721	1.53E-09	3.19E-06
*MT1F*	4.624068	1.71E-08	2.38E-05
*IFIT3*	3.159116	3.43E-08	3.40E-05
*MT2A*	3.897841	6.35E-08	4.68E-05
*HERC5*	3.679789	2.13E-07	0.000148
*OASL*	4.037971	3.34E-07	0.000199
*CXCL1*	-2.70168	3.84E-07	0.000219
*MT2P1*	3.396589	7.79E-07	0.000375
*RTP4*	2.2891	1.07E-06	0.00048
*TRIB3*	1.753461	1.96E-06	0.000765
*SDS*	1.96803	2.65E-06	0.001005
*PROC*	2.24197	3.93E-06	0.00125
*CMPK2*	2.977134	4.73E-06	0.001378
*MT1XP1*	3.749513	4.87E-06	0.001386
*LZTS1*	-2.51467	6.31E-06	0.001718
*DDX58*	2.437334	6.63E-06	0.001766
*BATF3*	1.5738	0.000391	0.03548
*KCNK5*	-1.82565	0.000453	0.0394
*LURAP1L*	1.643754	0.00048	0.040877
*OAS1*	1.576962	0.00049	0.040918

### GO, KEGG and SPIA enrichment analysis for DEGs downstream empty vector

**Figure 6:**
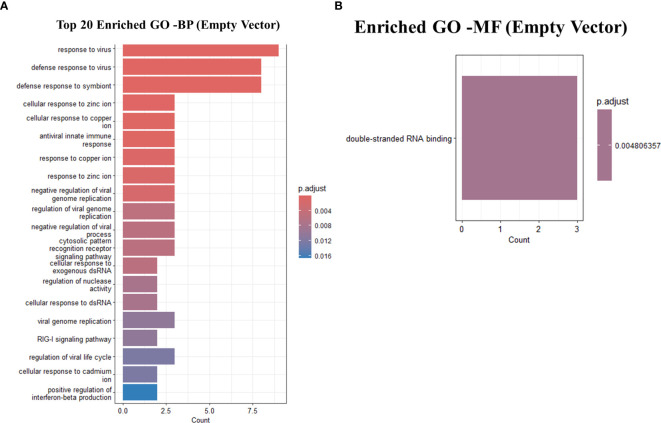
GO analysis showing only the top 20 significantly enriched pathways in Empty-vector only transfected THP-1 infected cells with R179*M. tb*. **(A)** GO enriched terms for BP **(B)** GO enriched terms for MF. The color of the bars indicates significance (adjusted P-value), and the size corresponds to the count of genes.

Predictably, the GO terms were primarily enriched in response to virus, defense response to virus, defense response to symbiont, cellular response to zinc ion, cellular response to copper ion, antiviral innate immune response, response to copper ion, response to zinc ion, negative regulation of viral genome replication, regulation of viral genome replication, negative regulation of viral process, cytosolic pattern recognition receptor signaling pathway, cellular response to exogenous dsRNA, regulation of nuclease activity (**Figure 6**). A table showing the top 15 GO terms and all KEGG with associated gene lists and statistical values can be found in **Tables 7**, **8**.

**Table 7:** Top 15 enrichment GO terms for Biological Process (BP) in THP-1 cells infected with R179*M.tb* after transfection with empty vector only with their corresponding DEGs involved.

GOBP ID	Description	P. Adjusted Value	Gene ID
GO:0009615	response to virus	1.12E-07	*IFIT2/IFIT1/IFIT3/HERC5/OASL/RTP4/RIGI/BATF3/OAS1*
GO:0051607	defense response to virus	1.30E-07	*IFIT2/IFIT1/IFIT3/HERC5/OASL/RTP4/RIGI/OAS1*
GO:0140546	defense response to symbiont	1.30E-07	*IFIT2/IFIT1/IFIT3/HERC5/OASL/RTP4/RIGI/OAS1*
GO:0071294	cellular response to zinc ion	0.000132	*MT1E/MT1F/MT2A*
GO:0071280	cellular response to copper ion	0.000234	*MT1E/MT1F/MT2A*
GO:0140374	antiviral innate immune response	0.000234	*IFIT1/RIGI/OAS1*
GO:0046688	response to copper ion	0.000813	*MT1E/MT1F/MT2A*
GO:0010043	response to zinc ion	0.001077	*MT1E/MT1F/MT2A*
GO:0045071	negative regulation of viral genome replication	0.00154	*IFIT1/OASL/OAS1*
GO:0045069	regulation of viral genome replication	0.0051	*IFIT1/OASL/OAS1*
GO:0048525	negative regulation of viral process	0.005291	*IFIT1/OASL/OAS1*
GO:0002753	cytosolic pattern recognition receptor signaling pathway	0.005291	*OASL/RIGI/OAS1*
GO:0071360	cellular response to exogenous dsRNA	0.005291	*IFIT1/RIGI*
GO:0032069	regulation of nuclease activity	0.00776	*OASL/OAS1*

**Table 8:** All KEGG enriched pathways in THP-1 cells infected with R179*M.tb* after empty vector only transfection with their corresponding enrichment score.

KEGG ID	Description	Enrichment Score
hsa04978	Mineral absorption	0.904178
hsa04742	Taste transduction	-0.79694

The SPIA results for the empty vector, as shown in **Table 9**, **Supplementary Figure 6**, revealed intriguing findings that underscore the distinct effectiveness of IFIT2 induction in anti-mycobacterial activity compared to the empty vector. Notably, the analysis demonstrated that pathways such as the cytokine-cytokine mediated interaction pathway, chemokine signaling pathway, and apoptosis were inhibited by the empty vector but were activated downstream by IFIT2 induction.

**Table 9:** Top 15 Pathways enriched for SPIA analysis after empty vector transfection in THP-1 cells infected with R179*M.tb* and their corresponding status.

Pathway ID	Pathway	pPERT	pGFdr	Status
4060	Cytokine-cytokine receptor interaction	0.014	5.90E-05	Inhibited
5164	Influenza A	0.075	0.001041403	Activated
4623	Cytosolic DNA-sensing pathway	0.03	0.008402814	Activated
5168	Herpes simplex infection	0.046	0.015206414	Activated
5323	Rheumatoid arthritis	0.271	0.016358433	Activated
5162	Measles	0.073	0.023851615	Activated
5160	Hepatitis C	0.58	0.024252426	Activated
4622	RIG-I-like receptor signaling pathway	0.146	0.024655959	Activated
4978	Mineral absorption	1	0.034865963	Inhibited
4064	NF-kappa B signaling pathway	0.153	0.037906359	Activated
5120	Epithelial cell signaling in Helicobacter pylori infection	0.445	0.037906359	Inhibited
4010	MAPK signaling pathway	0.75	0.096624766	Inhibited
5132	Salmonella infection	0.699	0.099246083	Inhibited
4150	mTOR signaling pathway	0.344	0.147441236	Activated
4062	Chemokine signaling pathway	0.026	0.148894006	Inhibited

### Comparing the transcriptomic profiles of *IFIT2*, empty vector and *CKB*

In comparison to *IFIT2*, the treatment with empty vector and *CKB* which served as controls for the present study revealed unexpected results in their transcriptomic profiles. To identify the commonalities and potential specific genes to each treatment, Venny online software v 2.1 (https://bioinfogp.cnb.csic.es/tools/venny/) was used to obtain the intersection of *IFIT2*/empty vector, *IFIT2*/*CKB*, and *IFIT2*/empty vector/*CKB*. Venn diagrams showing number and percentage of overlapping genes and genes exclusive to *IFIT2 M. tb* infected THP-1 cells can be found in **Supplementary Figure 7**. For *IFIT2*/empty vector comparison, the result showed that 20 genes were common to both, 50 genes were exclusive to *IFIT2* and 3 genes exclusive to Empty Vector (**Supplementary Figure 7A**)*. IFIT2*/*CKB* comparison results also revealed that 50 genes overlapped, 20 genes were *IFIT2* specific genes and 5 were specific to *CKB* (**Supplementary Figure 7B**). Lastly, for the three comparisons, the results showed that there were 20 DEGs common to all the three groups, 20 specific genes in *IFIT2*, 3 specific genes in Empty Vector and 3 specific genes for *CKB* (**Supplementary Figure 7C**).

### Protein-protein interaction networks

**Figure 7:**
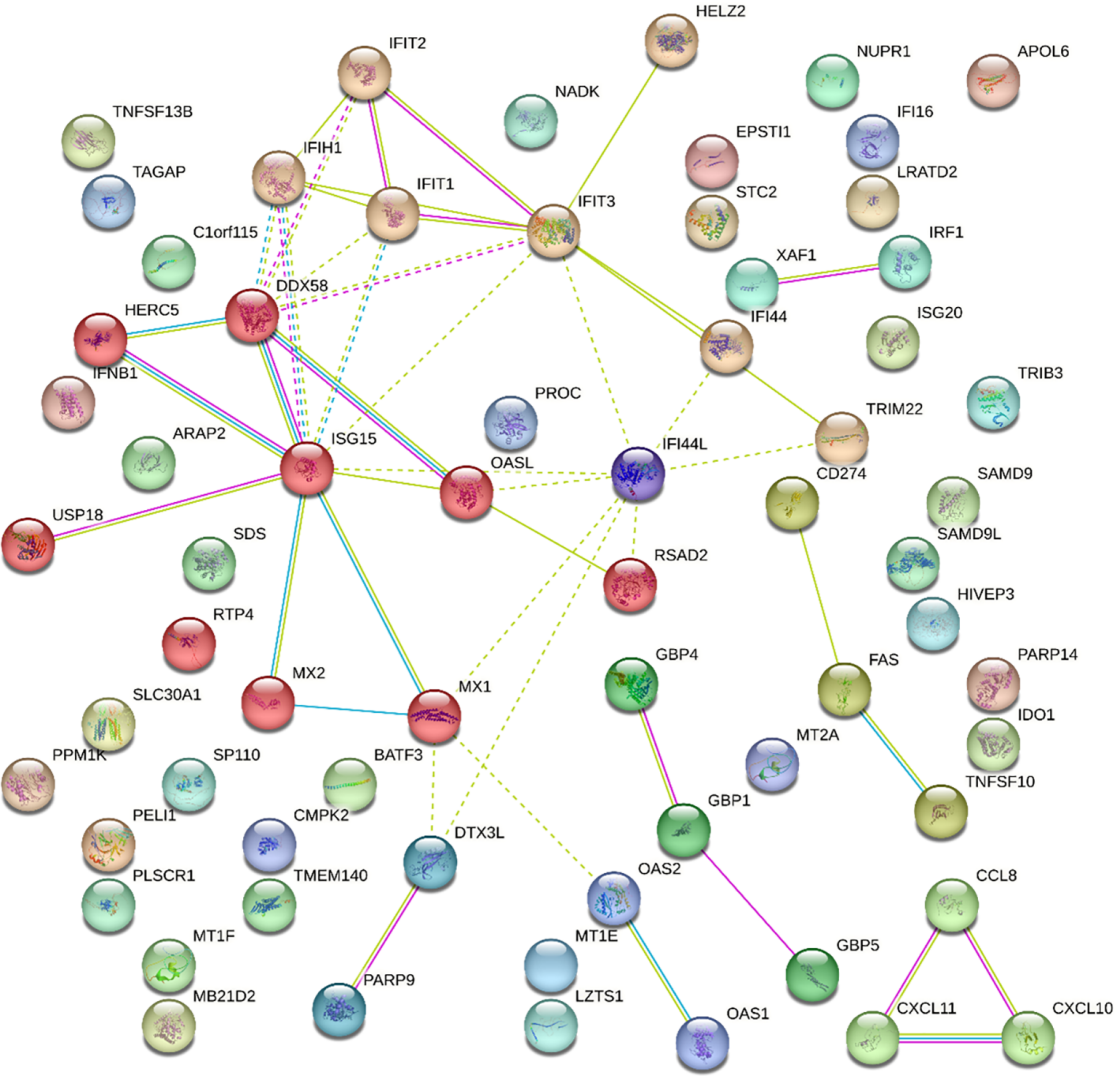
Protein-Protein Interactions of DEGs with p-adjusted values <0.05 and absolute log_2_(fold change) >1.5 for *IFIT2*. This is a STRING-generated physical subnetwork (the edges indicate that the proteins are part of a physical complex). Nodes are colored based on the clustering using the Markov Cluster Algorithm (MCL) option of STRING version 12.0.

A protein-protein interaction (PPI) network was constructed using the STRING database to assess functional associations among the proteins encoded by the DEGs of IFIT2 induction in *M. tb*-infected THP-1 cells. Each node represented a DEG, while connections between nodes indicated established interactions (**Figure 7**). This analysis provided insight into the functional relationships among the DEGs, enhancing our understanding of their roles in TB infection. Within the PPI network, several hub genes were identified based on their high degree of connectivity. Key hub genes include ISG15, RSAD2, IFIT3, IFI44L, suggesting their central roles in mediating interactions among DEGs induced by *M. tb* infection.

### RT-qPCR validation of selected DEGs

**Figure 8:**
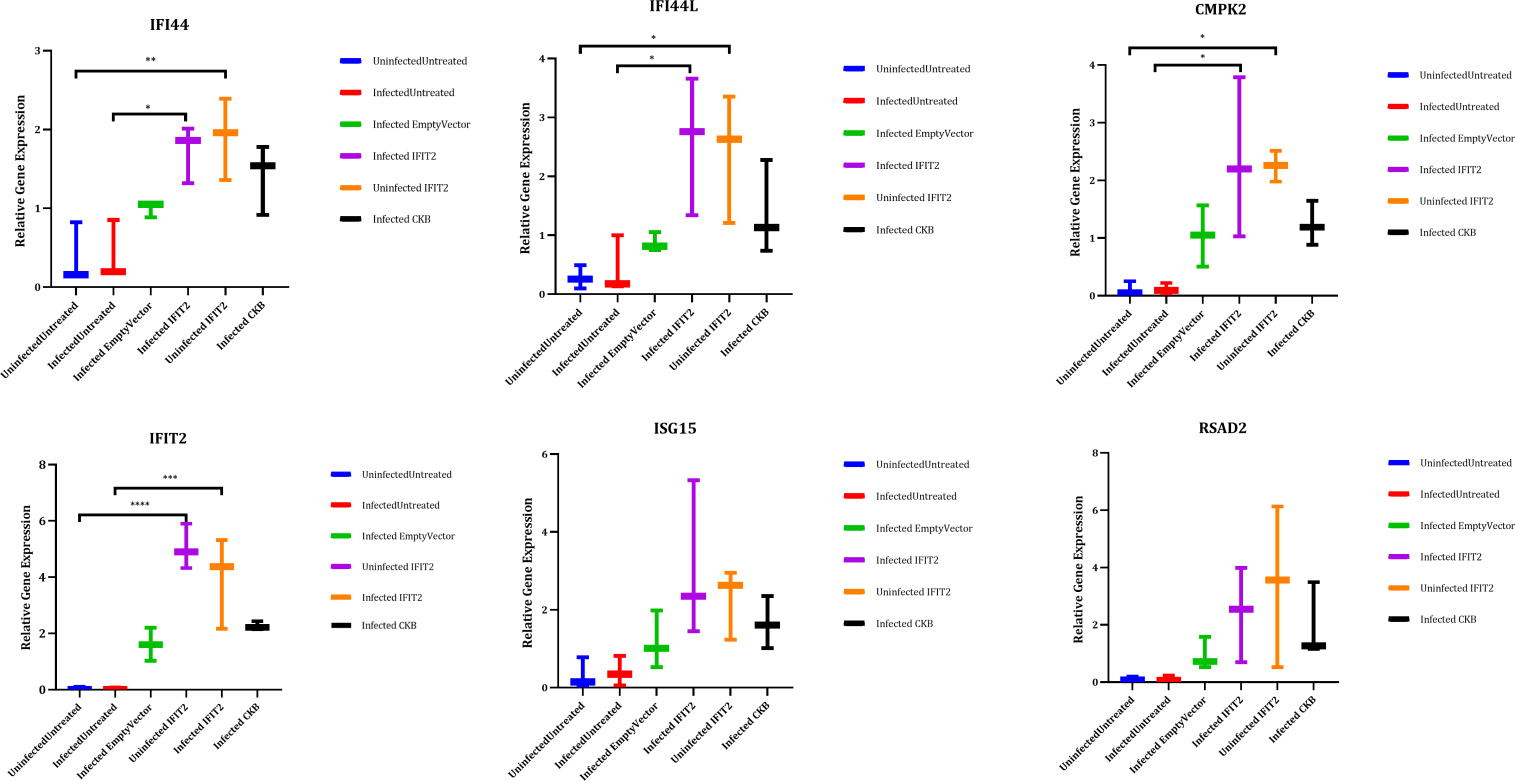
Validation of the selected genes by qPCR. IFIT2 expression was induced in THP-1 macrophages infected with *M. tb* or left uninfected, followed by RNA extraction. Three biological replicates were run in triplicates for each sample and analyzed by Roche Light Cycler 96 software to obtain the relative expression in relation to the reference genes UBC and GAPDH. One-way ANOVA with Tukey’s post-test was used to determine significance. *p-value < 0.05, **p-value <0.01, ***p-value <0.001, ****p-value <0.0001. GAPDH (Glyceraldehyde-3-phospate dehydrogenase), UBC (Ubiquitin C), IFIT2 (Interferon induced protein with tetratricopeptide repeat 2), ISG15 (interferon stimulated 15), IFI44L (Interferon-Induced Protein 44-Like), RSAD2 (Radical S-adenosyl methionine domain containing 2), CMPK2 (Cytidine/uridine monophosphate kinase 2), IFI44 (Interferon-Induced Protein 44).

Gene expression levels were quantified using RT-qPCR for five selected key DEGs identified downstream of IFIT2 which includes, CMPK2, RSAD2, ISG15,IFI44L and IFI44. The qPCR results were consistent with the AmpliSeq data, demonstrating robust fold changes and statistical significance (p < 0.05) albeit not for ISG15 and RSAD2 (**Figure 8**). This concordance reinforces the reliability of our transcriptomic analysis and highlights the potential role of these interferon-stimulated genes (ISGs) in the antimycobacterial activity of IFIT2. The enhanced expression of these genes suggests their potential contribution to the host’s immune defense mechanisms, offering promising targets for future therapeutic strategies against TB.

## Discussion

With the rise of drug-resistant TB, there is a growing interest in targeting host genes for improved therapy due to the less likelihood of host resistance developing. Recent studies have highlighted the potential role of the IFIT protein family in mycobacterial killing. Among them, IFIT2 stands out for its unique ability to inhibit cancer cell proliferation, making it a promising candidate for further research against *M. tb*. In this study, we explored the transcriptomic profile of the human IFIT2 gene induced in macrophages infected with *M. tb*, to elucidate its underlying anti-mycobacterial mechanisms.

Preliminary experiments to determine the best IFIT2 vector construct for our high-throughput experiments investigated the impact of the FLAG tag position (N-terminus or C-terminus) on IFIT2’s expression and anti-mycobacterial activity. Our findings revealed that the C-Flagged IFIT2 vector construct recorded high CFU counts and low IFIT2 gene expression levels. This is consistent with previous studies that observed placing a tag at the C-terminus can compromise protein stability and function. Studies on other proteins, such as the molybdoenzyme YedY (Sabaty et al., 2013) and antiviral gene viperin (Jiang et al., 2008), have shown similar detrimental effects of C-terminal tagging on enzymatic activity and antiviral function, respectively. Conversely, both the N-Flagged and No-Flag IFIT2 vector constructs showed high gene expression and significant anti-mycobacterial activity, indicating that the N-terminal tag or its absence did not hinder IFIT2’s functionality. These results support the preference for N-terminal tags in literature highlighting their advantages over C-terminal tags, which includes efficient translation efficiency and ease of tag removal from the target protein enhancing protein stability (Malhotra, 2009). Studies with other proteins, like recombinant antibody fragments in *E. coli* using antibody M1 (Plückthun, 1992) and Cytochrome P450 119 (CYP119) (Aslantas and Surmeli, 2019), have demonstrated improved expression levels and stability when tagged at the N-terminus compared to the C-terminus. Our present study reinforces that N-terminal tagging is advantageous over C-terminal tagging for maintaining protein integrity and function.

Our transcriptomic and bioinformatic analyses identified a comprehensive set of differentially expressed genes (DEGs) and associated pathways, revealing the complex interactions between host genes and mycobacterial infection. Notably, many of the significant DEGs participate in type I interferon (IFN) signaling, which is crucial for antiviral defense and immune response. Previous studies have established that type I IFNs are typically activated by mycobacterial infection (Akter et al., 2022; Madden et al., 2023). Downstream of IFIT2 induction, we found several interferon-stimulated genes (ISGs) such as IFIT1, IFIT3, OASL, OAS1, ISG15, IFI44L, MX1, MX2, RSAD2, CMPK2, and HERC5 upregulated. Madhvi et al. (2020) reported similar upregulation of these genes post *M. tb* infection in *M. tb*-infected hMDMs (Madhvi et al., 2020), however it is important to note that our study revealed higher log_2_(fold change) in IFIT2-induced R179 *M. tb*-infected THP-1 cells compared to R179 *M. tb*-infected hMDMs without IFIT2 induction. This suggests a direct association between IFIT2’s induction and the observed higher gene expressions of these ISGs. The higher fold changes in these ISGs likely contributed to IFIT2’s anti-mycobacterial effects, though this warrants further investigation. Interestingly, all these genes were found upregulated downstream of *IFIT2* in uninfected macrophages (with *IFIT2* induction) confirming that the induction of *IFIT2* leading to the upregulation of these genes could be independent of *M. tb* infection.

Additionally, several downstream genes identified in this study have documented roles in restricting mycobacterial growth. For example, the knock-up of interferon-induced protein 44-like (IFI44L) reduced mycobacterial growth after 24 hours, while its knock-down increased growth. Jiang et al. (2021) suggested that IFI44L promotes macrophage polarization and inflammatory cytokine secretion, inhibiting *M. tb* infection. Its upregulation was observed after rifampicin treatment, further restricting *M. tb* survival (Jiang et al., 2021). However, its homologous gene, IFI44, does not have a known role in TB infection but is shown to play a significant role in tumor cell recognition and immune response modulation. Furthermore, the silencing of oligoadenylate synthetase genes (OAS1, OAS2, OAS3) and oligoadenylate synthetase-like (OASL) was linked to increased mycobacterial growth, highlighting their potential roles in host defense (Leisching et al., 2017, 2019). Silencing the OASs significantly increases *M. tb* growth in THP-1 cells at 96 hours post-infection (Leisching et al., 2019). Previous transcriptomic studies revealed that these OASs distinguish latent TB from active TB (Berry et al., 2010; Ottenhoff et al., 2012). OASL expression is strongly induced by *M. tb* after 24 hours, and its knockdown led to an increase in *M. tb* after 96 hours (Leisching et al., 2017). Interferon regulatory factor 1 (IRF1) expression decreased mycobacterial survival in macrophages, playing a crucial role in *M. tb’s* intracellular survival, and is found to be high in TB patients compared to healthy controls (Zhou et al., 2019; Wu et al., 2021). Contrarily, Viperin (RSAD2) has been reported to increase *M. tb* infection in mice (Zhou et al., 2022). This may reflect a decrease in *M. tb* uptake rather than killing. Though the data showed a slight increase in *M. tb* infection with Viperin induction, the biological relevance remains uncertain. A research study found that viperin catalyzes the conversion of cytidine triphosphate to 3’-deoxy-3’,4’-didehydro-cytidine triphosphate, which acts as a chain terminator for RNA polymerases in viruses (Rivera-Serrano et al., 2017). CMPK2 plays a crucial role in nucleic acid synthesis in macrophages, with its expression induced by pattern-associated molecular patterns (PAMP) like lipopolysaccharide (LPS), a component of gram-negative bacterial outer membranes (Kim et al., 2021).

Our study also revealed the upregulation of chemokine-related genes including CXCL10, CXCL11, TNFSF10, TNFSF13B downstream of IFIT2. Previous research indicates that mycobacterial infection induces the release of chemokines like CXCL10 and CXCL11, crucial for immune mediator formation and granuloma development in TB-specific T cells (Domingo-Gonzalez et al., 2016). CXCL10 has been proposed as a potential TB biomarker, with its serum levels decreasing post-treatment compared to non-responders. CXCL10, though its exact role in TB remains unclear, has potential as a biomarker for monitoring TB treatment response (Almeida et al., 2009). Levels of CXCL10 and CXCL11 also distinguish TB infections from healthy controls (Sampath et al., 2023). TNFSF13B (B cell activating factor) is implicated in pulmonary diseases, promoting B cell maturation and proliferation (Fan et al., 2021). TNFSF10 shows increased expression during *M. tb* infection, yet its specific role remains uncertain (Badr and Häcker, 2019).

We also observed the upregulation of genes involved in ISGylation (ISG15, HERC5, USP18), a process where ISG15 is covalently attached to target proteins, enhancing host defense against intracellular pathogens. ISGylation has been shown to protect mice against *M. tb* (Kimmey et al., 2017), with ISG15 deficiency in humans linked to increased mycobacterial susceptibility (Bogunovic et al., 2012). A study found that HERC5-mediated ISGylation of the Phosphatase and Tensin Homolog (PTEN) activated the P13K-ART signaling pathway, causing proinflammatory cytokine production and promoting *M. tb* clearance (Du et al., 2023). USP18 on the other hand reverses ISGylation by detaching ISG15 from target proteins (Malakhov et al., 2002). Its expression was observed to increase during *M. tb* infection, and a study found that USP18 levels significantly rise in TB patients after isoniazid (INH) treatment, suggesting its potential as a marker for monitoring therapy response (De Oyarzabal et al., 2019).

We also identified apoptosis-related genes downstream of IFIT2 in *M. tb*-infected macrophages, including FAS, XAF1, TNFSF10, and TNFSF13B. Consistent with previous data, TNFSF10 and XAF1 were upregulated in T cells of active TB patients compared to healthy individuals (Wang et al., 2023). Research indicates that the FAS receptor’s interaction with its ligand activates caspase-8 (CASP8) through the Fas-associated death domain (FADD), leading to apoptosis. Previous studies have already established the involvement of *IFIT2* in apoptosis and attributed this to its ability to inhibit cancer cell proliferation (Ohsugi et al., 2017). However, the direct link between IFIT2’s apoptotic effects and anti-mycobacterial activity requires experimental validation. XAF1 promotes apoptosis through p53 (Wang et al., 2023) while MT1F, a tumor suppressor, inhibits cancer cell migration and invasion (Yan et al., 2012). Among highly expressed genes with log_2_(fold change) > 1.5, only two genes, HIVEP3 and LZTS1, were found downregulated downstream of IFIT2. HIVEP3, a transcription factor, binds to genes involve in cell progression and differentiation (Allen et al., 2002), while LZTS1 acts as a tumor suppressor (Zhou et al., 2015). Their downregulation suggests potential impacts on immune regulation, underscoring the need for further research into their specific roles in mycobacterial infection.

The significant enrichment of Gene Ontology (GO) terms related to “defense response to virus” and “cytokine-mediated signaling pathway” suggests that upregulated DEGs contribute to a broad spectrum of immune responses. Functional analysis revealed involvement in crucial pathways such as cytokine-cytokine receptor interaction and Jak-STAT signaling, essential for sustaining immune responses against intracellular pathogens. KEGG pathway analysis supported these findings, indicating significant impacts on these pathways and inhibition of the tuberculosis pathway, highlighting IFIT2’s role in enhancing immune responses to eliminate *M. tb*. Protein-protein interaction network analysis identified key hub genes (ISG15, RSAD2, IFI44L, IFIT3) central to the IFIT2-mediated immune response, suggesting coordinated efforts to enhance macrophage function. RT-qPCR validation of key DEGs (ISG15, CMPK2, RSAD2, IFI44L, IFI44) confirmed the reliability and credibility of our AmpliSeq data, reinforcing the biological relevance of identified genes and their roles in TB.

An interesting finding from this study is the observed anti-mycobacterial activity and IFIT2 gene expression in the control groups, specifically the empty vector and the non-specific gene vector (CKB). Our transcriptomic analysis revealed that the empty vector induced a non-specific antiviral response in the host cell by upregulating genes known as the metallothioneins including MT1E, MT1F, MT2A, MT2P1. These genes are involved in copper (Cu) and zinc (Zn) homeostasis, which play crucial roles in innate and adaptive immunity (Vignesh and Deepe, 2017). Zinc deficiency has been linked to impaired immune cell production and increased susceptibility to infections (Haase and Rink, 2014), while excess zinc can be cytotoxic (Shankar and Prasad, 2018). Though the precise roles of metallothioneins in TB immunity are not well established, they likely regulate host immunity. During TB infection, human macrophages deliver zinc into phagolysosomes to intoxicate the pathogen, contrary to the traditional belief in metal starvation as an antimicrobial defense (Botella et al., 2011). Additionally, the empty vector significantly upregulated several interferon-stimulated genes (ISGs) with known antiviral functions, including IFIT1, IFIT2, IFIT3, HERC5, OASL and RTP4. This aligns with previous findings that empty vectors can trigger cellular responses resembling viral infections (Hagen et al., 2015). These data support the hypothesis that empty vectors elicit a non-specific antiviral response, leading to observed anti-mycobacterial effects and the induced expression of the antiviral gene IFIT2.

## Conclusion

This study provides valuable insights into the transcriptomic profile of IFIT2-induced expression in TB, highlighting the downstream contributors and signaling pathways involved in the intracellular killing of *M. tb*. The findings underscore the potential of IFIT2 as a therapeutic target, paving the way for future research to exploit its immune-modulating properties for TB treatment. The upregulation of immune-related genes and inhibition of TB pathways suggest that enhancing IFIT2 expression or mimicking its effects could provide new treatment avenues. By advancing our understanding of host-pathogen interactions, this study contributes to the ongoing efforts to develop more effective TB therapies. Future research should explore the therapeutic potential of IFIT2, including the development of small molecules to modulate its activity. Broader transcriptomic and proteomic analyses, involving multiple cell types and *in vivo* models, are necessary to validate and extend these findings, providing a comprehensive understanding of host-pathogen interactions downstream of IFIT2. This will pave the way for innovative TB treatments leveraging host-targeted strategies to combat drug-resistant strains.

## Data Availability

Data generated and analyzed during this study have been deposited in the NCBI Gene Expression Omnibus (GEO) under accession number GSE297207.

## Supplementary material

Supplementary Figure 1 Knock-up of *IFIT2* using different vector constructs (N-Flagged *IFIT2*, C-Fagged *IFIT2*, and No-Flag *IFIT2*) in *M. bovis* BCG-infected THP-1 cells assessed by CFUs and relative gene expression. No-flag 1 and No-flag 2 represent technical replicates **(A)** Intracellular mycobacteria via CFU counts at 24 h post-infection. **(B)** Relative mRNA expression levels of *IFIT2*. Included in the graph are the statistical significance (ANOVA and Tukey post-test) levels for the indicated vector treatments against the 24 h infected control. *p-value < 0.05, **p-value <0.01, ***p-value <0.001, ****p-value < 0.0001. TR: Transfection Reagent, ns: not significant.

Supplementary Figure 2 Knock-up of *IFIT2* using different vector constructs (N-Flagged *IFIT2* and No-Flag *IFIT2*) in R179 *M.tb*-infected THP-1 cells assessed by CFUs and relative gene expression. No-flag 1 and No-flag 2 represent technical replicates **(A)** Intracellular mycobacteria via CFU counts at 24 h post-infection. **(B)** Relative mRNA expression levels of *IFIT2* in various samples. Included in the graph are the statistical significance (ANOVA and Tukey post-test) levels for the indicated vector treatments against the 24hours infected control. *p-value < 0.05, **p-value <0.01, ***p-value <0.001. TR, Transfection Reagent; ns, not significant; CKB, creatine kinase B (non-specific vector).

Supplementary Figure 3 MA plot showing the log-fold change versus the mean expression for the treatment groups. **(A)** IFIT2-transfected THP-1 cells infected with R179M.tb, **(B)** Empty Vector-transfected THP-1 cells infected with R179M.tb, and **(C)** CKB-transfected THP-1 cells infected with R179M.tb) compared to control (R179M.tb-infected THP-1 cells only). Blue dots indicate significant differentially expressed genes (DEGs); false discover rate (FDR) <0.05.

Supplementary Figure 4 Upsetplot for Enriched KEGG pathways in DEGs (in THP-1 cells infected with R179*M.tb* after IFIT2 induction. Each dot represents a gene. The plot shows unique genes to specific pathways and their overlaps in different pathways.

Supplementary Figure 5 SPIA two-way evidence plot for THP-1 cells infected with R179M.tb with the induced expression of IFIT2. SPIA of all DEGs based on pathway gene enrichment (pNDE) and pathway perturbations (pPERT) considering gene placement and topology within the pathway. Both pNDE and pPERT are used to determine global significance. Each dot represents a pathway that contains at least one DEG. The impact analysis plots each pathway based on pNDE and pPERT. Pathways above the solid blue line are significant following FDR correction (pGFDR < 0.05). Pathways above the solid red line are significant following Bonferroni correction (pGFWER < 0.05). The pathways associated with the pathway IDs in this figure can be found in **Table 5**.

Supplementary Figure 6 SPIA two-way evidence plot for THP-1 cells infected with R179*M.tb* after transfecting with empty vector. SPIA of all DEGs based on pathway gene enrichment (pNDE) and pathway perturbations (pPERT) considering gene placement and topology within the pathway. Both pNDE and pPERT are used to determine global significance. Each dot represents a pathway that contains at least one DEG. The impact analysis plots each pathway based on pNDE and pPERT. Pathways above the solid blue line are significant following FDR correction (pGFDR < 0.05). Pathways above the solid red line are significant following Bonferroni correction (pGFWER < 0.05). The pathways associated with the pathway IDs in this figure can be found in **Table 9**.

Supplementary Figure 7 Differential gene expression treatment comparisons by Venn diagram analysis. **(A)** The number of DEGs that overlap between *IFIT2 M. tb* infected THPS-1 cells vs *M. tb* Infected THP-1 cells **(B)** The number of DEGs that overlap between *IFIT2 M. tb* infected THP-1 cells vs *M. tb* Infected THP-1 cells **(C)** Number of differential gene expression patterns that overlap in all the various treatment groups.
