# Supplementary figures and images for: IFIT2-induced transcriptomic changes in Mycobacterium tuberculosis infected macrophages
Source: Front Cell Infect Microbiol. 2025 May 20;15:1536446. doi: 10.3389/fcimb.2025.1536446 (PMC12131013; doi:10.3389/fcimb.2025.1536446)

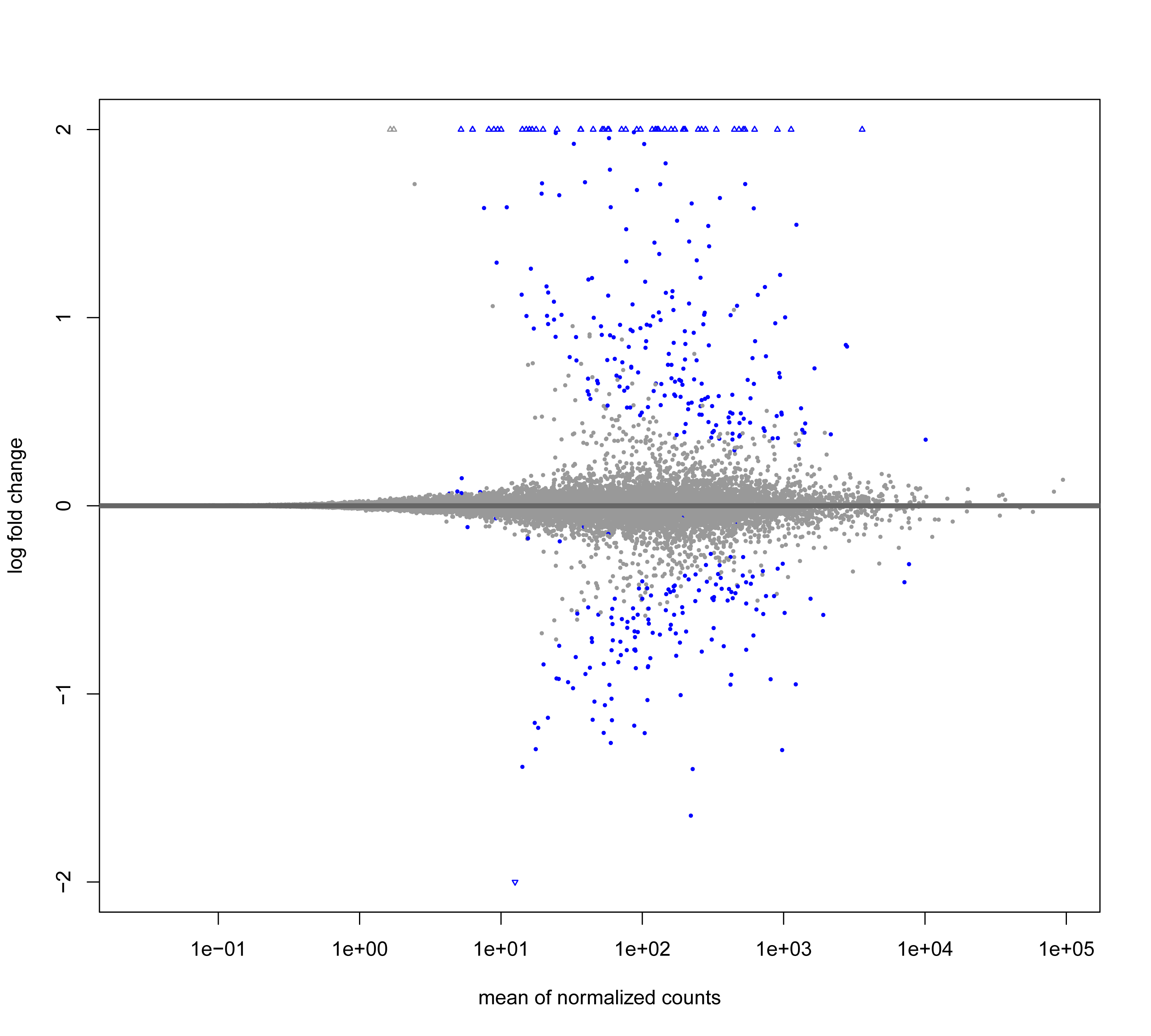

Supplement: Supplementary Figure 1 — Knock-up of IFIT2 using different vector constructs (N-Flagged IFIT2, C-Fagged IFIT2, and No-Flag IFIT2) in M. bovis BCG-infected THP-1 cells assessed by CFUs and relative gene expression. No-flag 1 and No-flag 2 represent technical replicates (A) Intracellular mycobacteria via CFU counts at 24 h post-infection. (B) Relative mRNA expression levels of IFIT2. Included in the graph are the statistical significance (ANOVA and Tukey post-test) levels for the indicated vector treatments against the 24 h infected control. *p-value < 0.05, **p-value <0.01, ***p-value <0.001, ****p-value < 0.0001. TR: Transfection Reagent, ns: not significant. [file DataSheet1.zip › Supplementary Figures/Supplementary Figure 3A.tif]

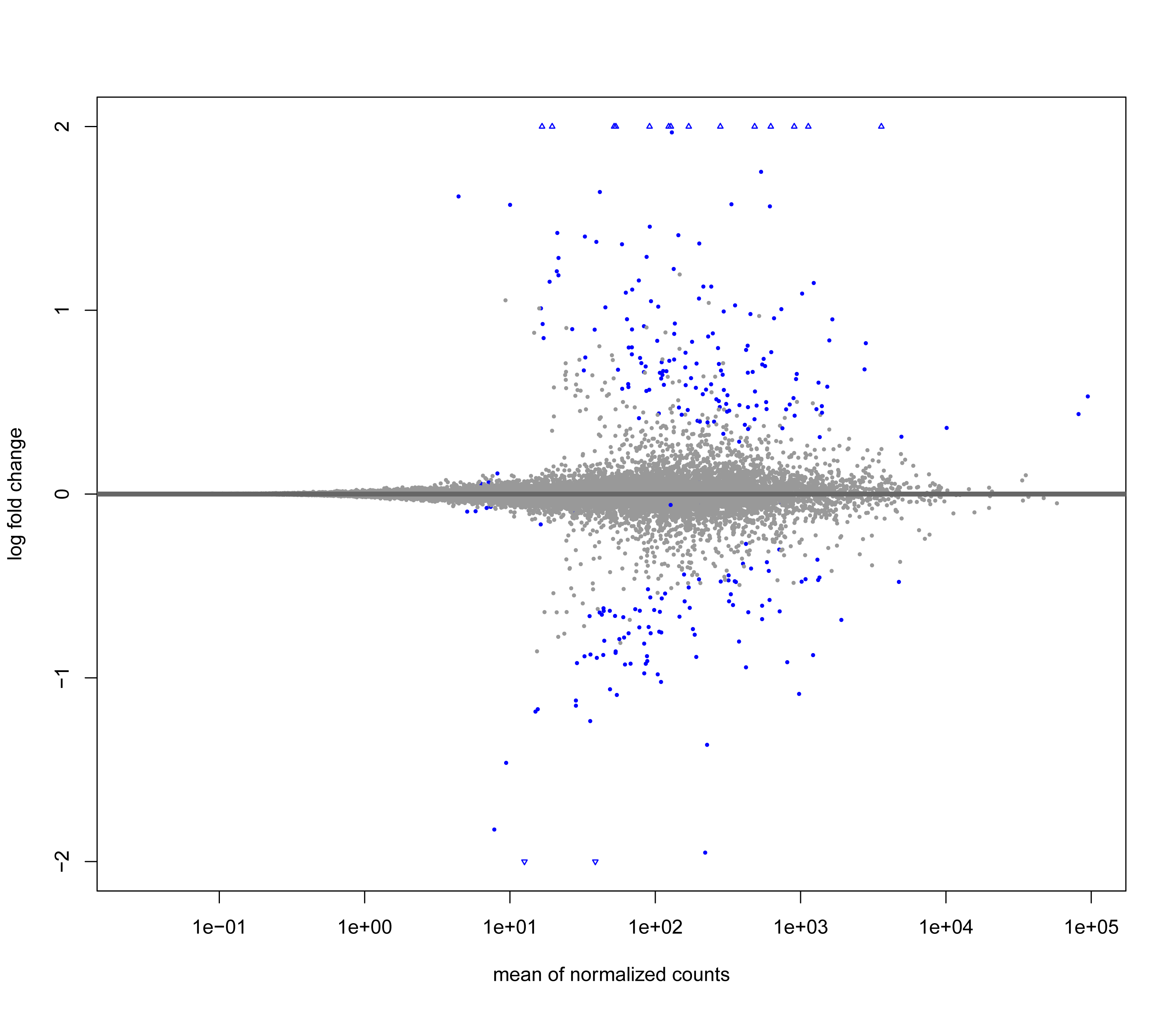

Supplement: Supplementary Figure 1 — Knock-up of IFIT2 using different vector constructs (N-Flagged IFIT2, C-Fagged IFIT2, and No-Flag IFIT2) in M. bovis BCG-infected THP-1 cells assessed by CFUs and relative gene expression. No-flag 1 and No-flag 2 represent technical replicates (A) Intracellular mycobacteria via CFU counts at 24 h post-infection. (B) Relative mRNA expression levels of IFIT2. Included in the graph are the statistical significance (ANOVA and Tukey post-test) levels for the indicated vector treatments against the 24 h infected control. *p-value < 0.05, **p-value <0.01, ***p-value <0.001, ****p-value < 0.0001. TR: Transfection Reagent, ns: not significant. [file DataSheet1.zip › Supplementary Figures/Supplementary Figure 3B.tif]

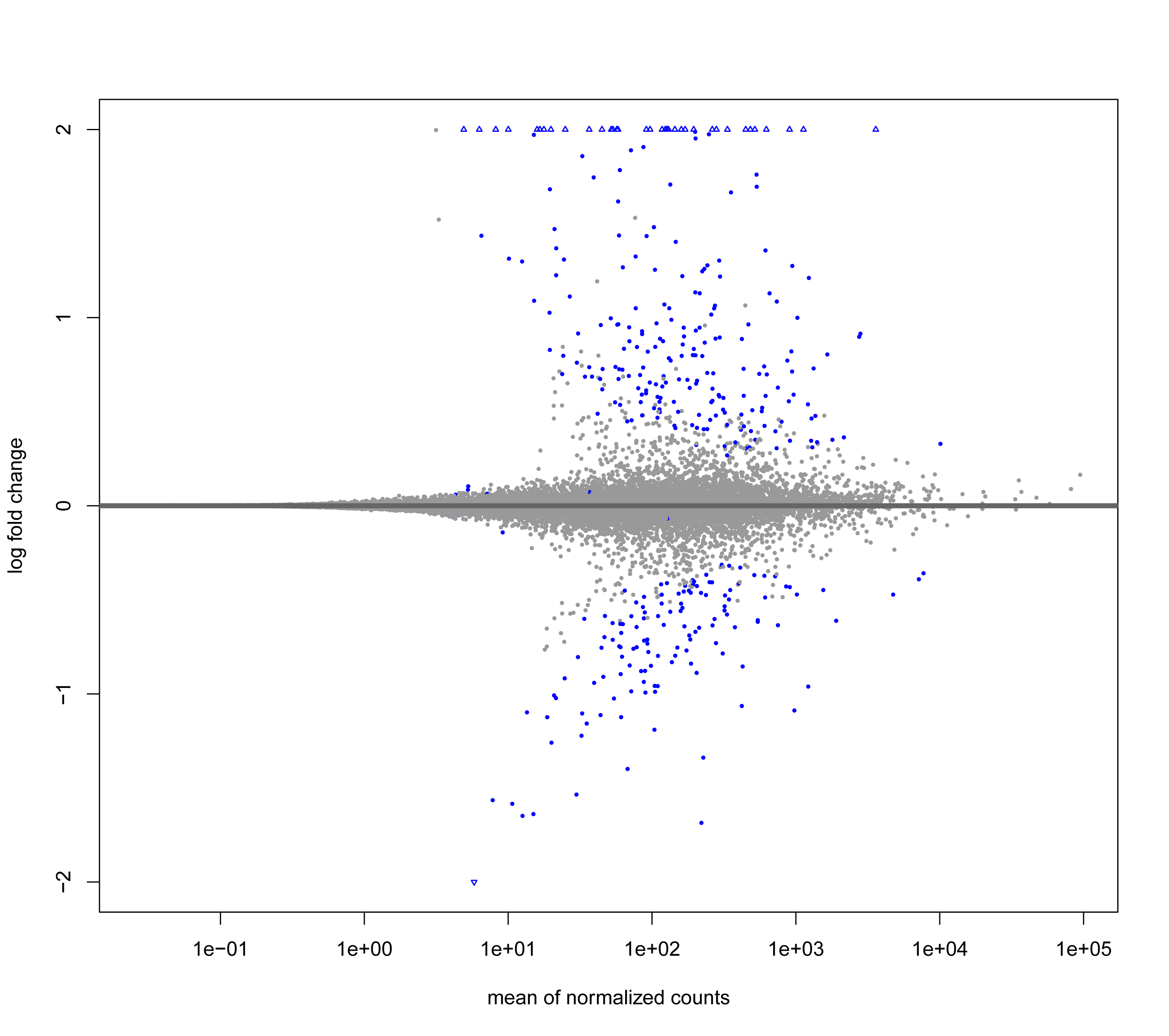

Supplement: Supplementary Figure 1 — Knock-up of IFIT2 using different vector constructs (N-Flagged IFIT2, C-Fagged IFIT2, and No-Flag IFIT2) in M. bovis BCG-infected THP-1 cells assessed by CFUs and relative gene expression. No-flag 1 and No-flag 2 represent technical replicates (A) Intracellular mycobacteria via CFU counts at 24 h post-infection. (B) Relative mRNA expression levels of IFIT2. Included in the graph are the statistical significance (ANOVA and Tukey post-test) levels for the indicated vector treatments against the 24 h infected control. *p-value < 0.05, **p-value <0.01, ***p-value <0.001, ****p-value < 0.0001. TR: Transfection Reagent, ns: not significant. [file DataSheet1.zip › Supplementary Figures/Supplementary Figure 3C.tif]

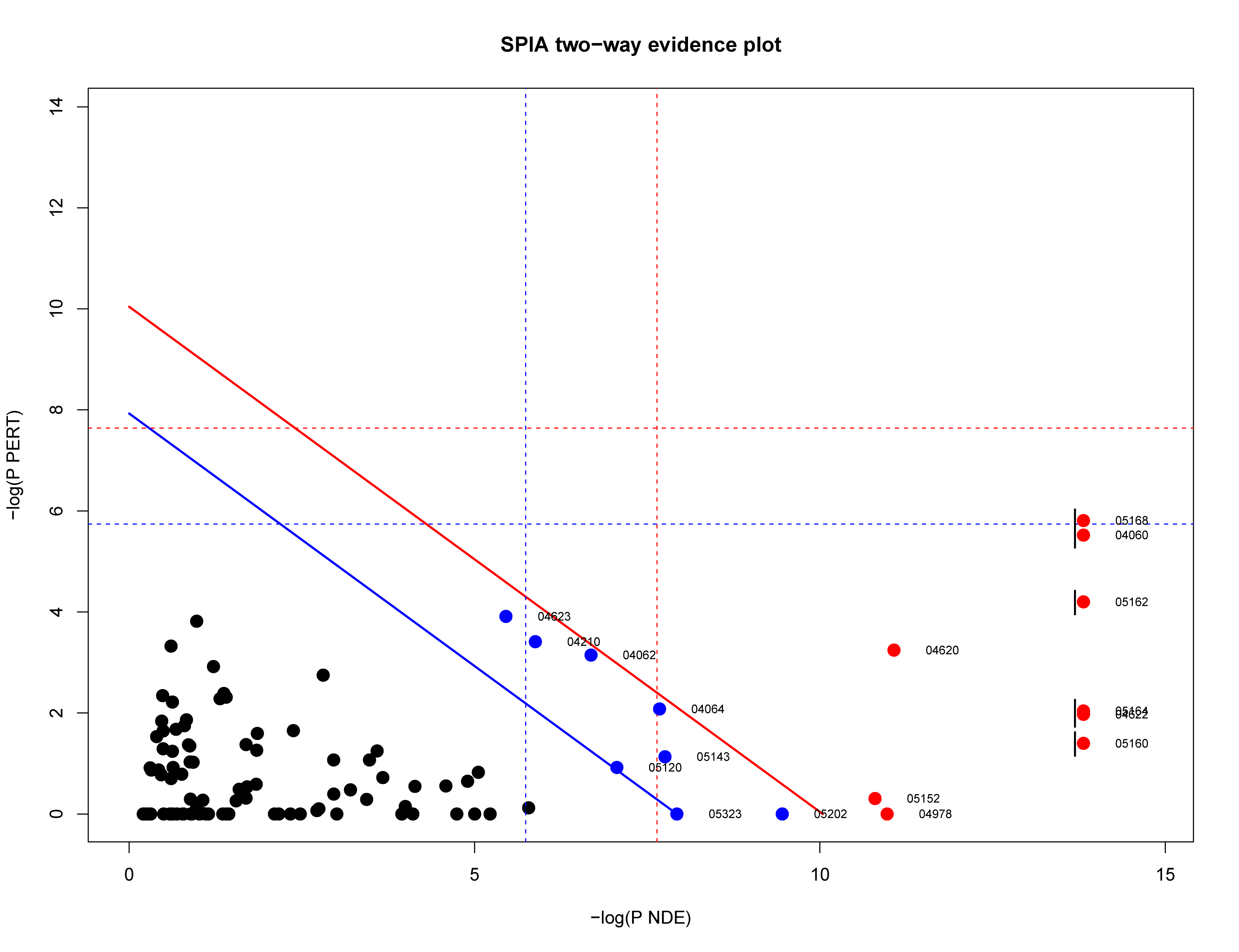

Supplement: Supplementary Figure 1 — Knock-up of IFIT2 using different vector constructs (N-Flagged IFIT2, C-Fagged IFIT2, and No-Flag IFIT2) in M. bovis BCG-infected THP-1 cells assessed by CFUs and relative gene expression. No-flag 1 and No-flag 2 represent technical replicates (A) Intracellular mycobacteria via CFU counts at 24 h post-infection. (B) Relative mRNA expression levels of IFIT2. Included in the graph are the statistical significance (ANOVA and Tukey post-test) levels for the indicated vector treatments against the 24 h infected control. *p-value < 0.05, **p-value <0.01, ***p-value <0.001, ****p-value < 0.0001. TR: Transfection Reagent, ns: not significant. [file DataSheet1.zip › Supplementary Figures/Supplementary Figure 5.tif]

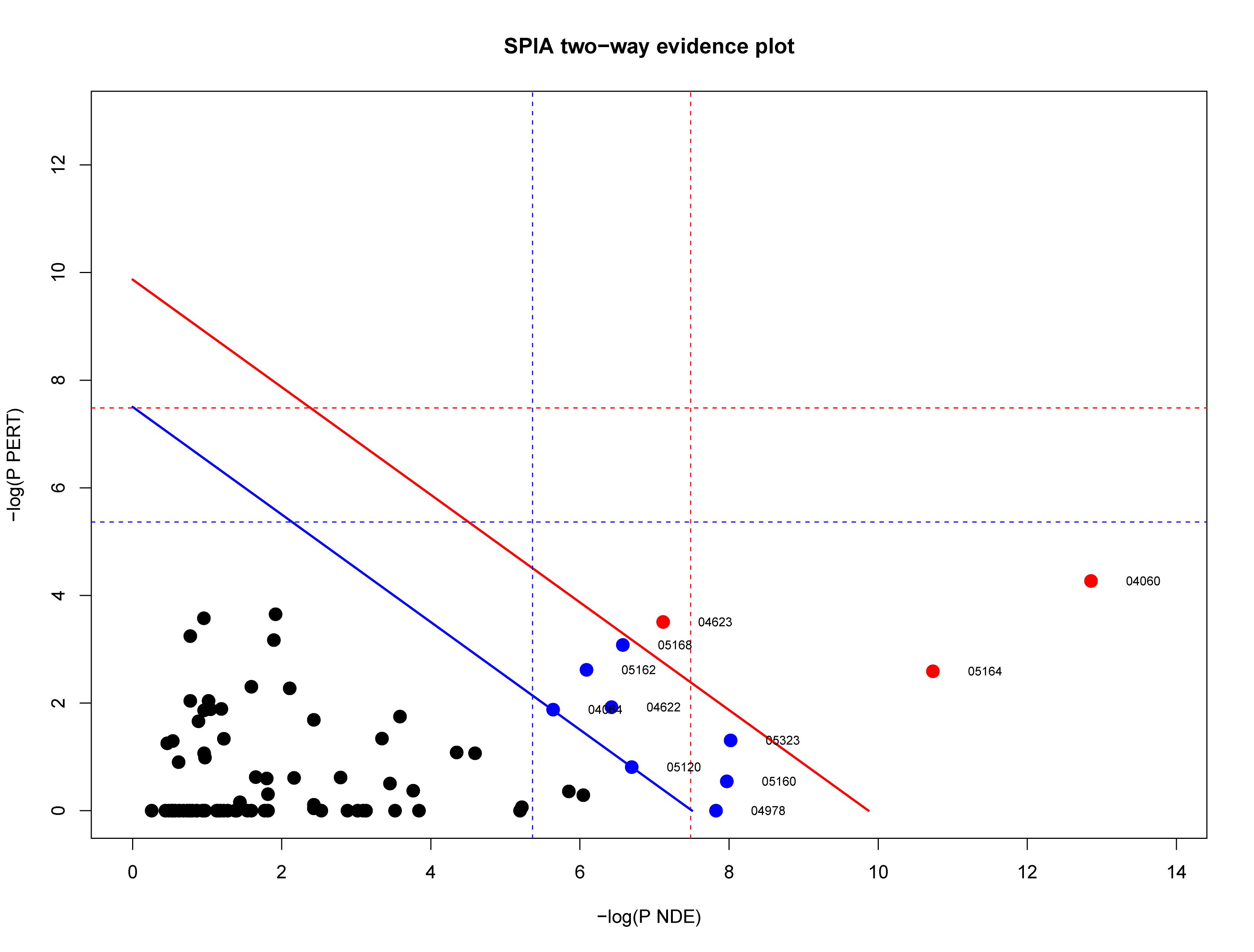

Supplement: Supplementary Figure 1 — Knock-up of IFIT2 using different vector constructs (N-Flagged IFIT2, C-Fagged IFIT2, and No-Flag IFIT2) in M. bovis BCG-infected THP-1 cells assessed by CFUs and relative gene expression. No-flag 1 and No-flag 2 represent technical replicates (A) Intracellular mycobacteria via CFU counts at 24 h post-infection. (B) Relative mRNA expression levels of IFIT2. Included in the graph are the statistical significance (ANOVA and Tukey post-test) levels for the indicated vector treatments against the 24 h infected control. *p-value < 0.05, **p-value <0.01, ***p-value <0.001, ****p-value < 0.0001. TR: Transfection Reagent, ns: not significant. [file DataSheet1.zip › Supplementary Figures/Supplementary Figure 6.tif]

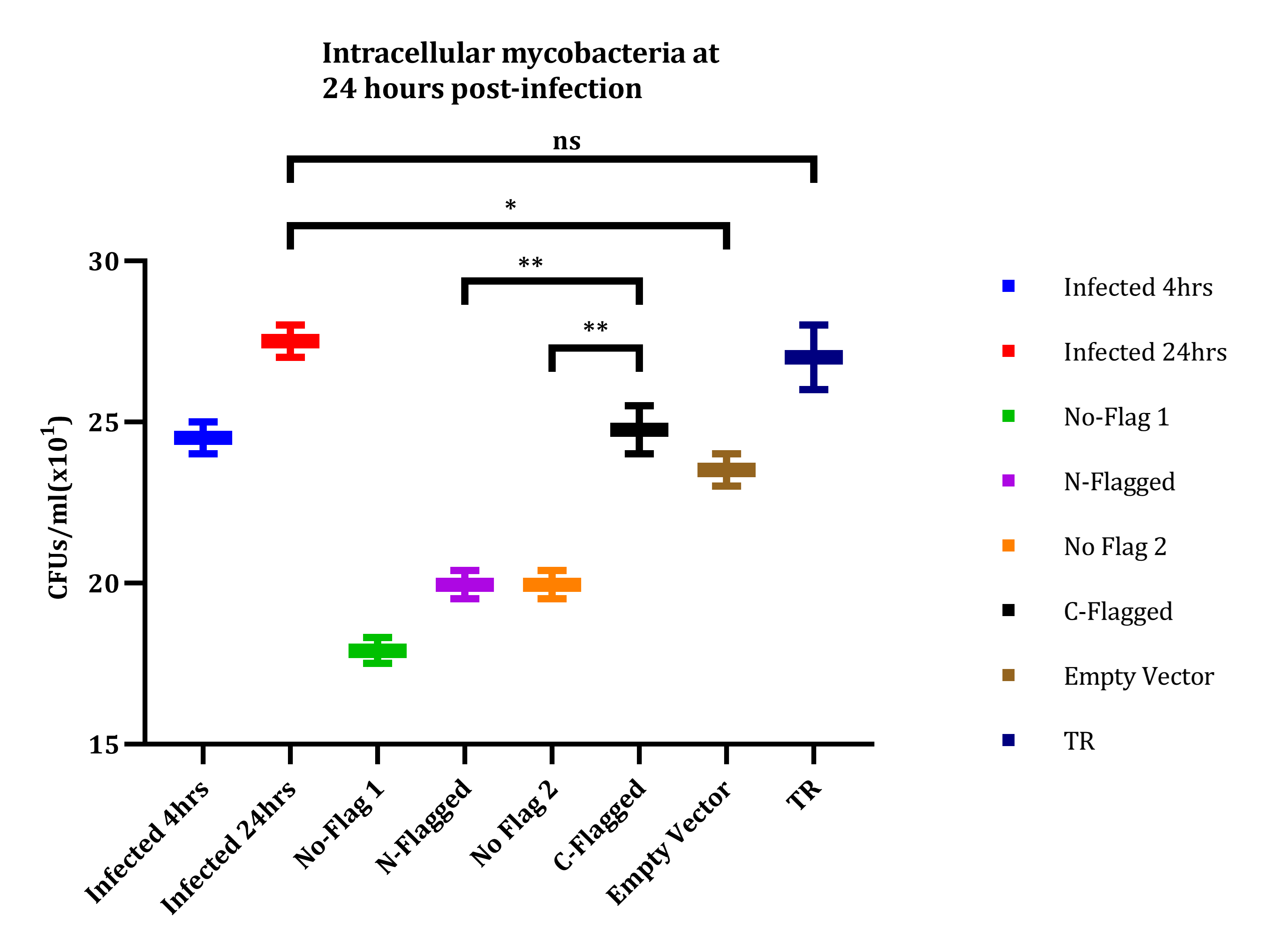

Supplement: Supplementary Figure 1 — Knock-up of IFIT2 using different vector constructs (N-Flagged IFIT2, C-Fagged IFIT2, and No-Flag IFIT2) in M. bovis BCG-infected THP-1 cells assessed by CFUs and relative gene expression. No-flag 1 and No-flag 2 represent technical replicates (A) Intracellular mycobacteria via CFU counts at 24 h post-infection. (B) Relative mRNA expression levels of IFIT2. Included in the graph are the statistical significance (ANOVA and Tukey post-test) levels for the indicated vector treatments against the 24 h infected control. *p-value < 0.05, **p-value <0.01, ***p-value <0.001, ****p-value < 0.0001. TR: Transfection Reagent, ns: not significant. [file DataSheet1.zip › Supplementary Figures/Supplementary Figure 1A.tif]

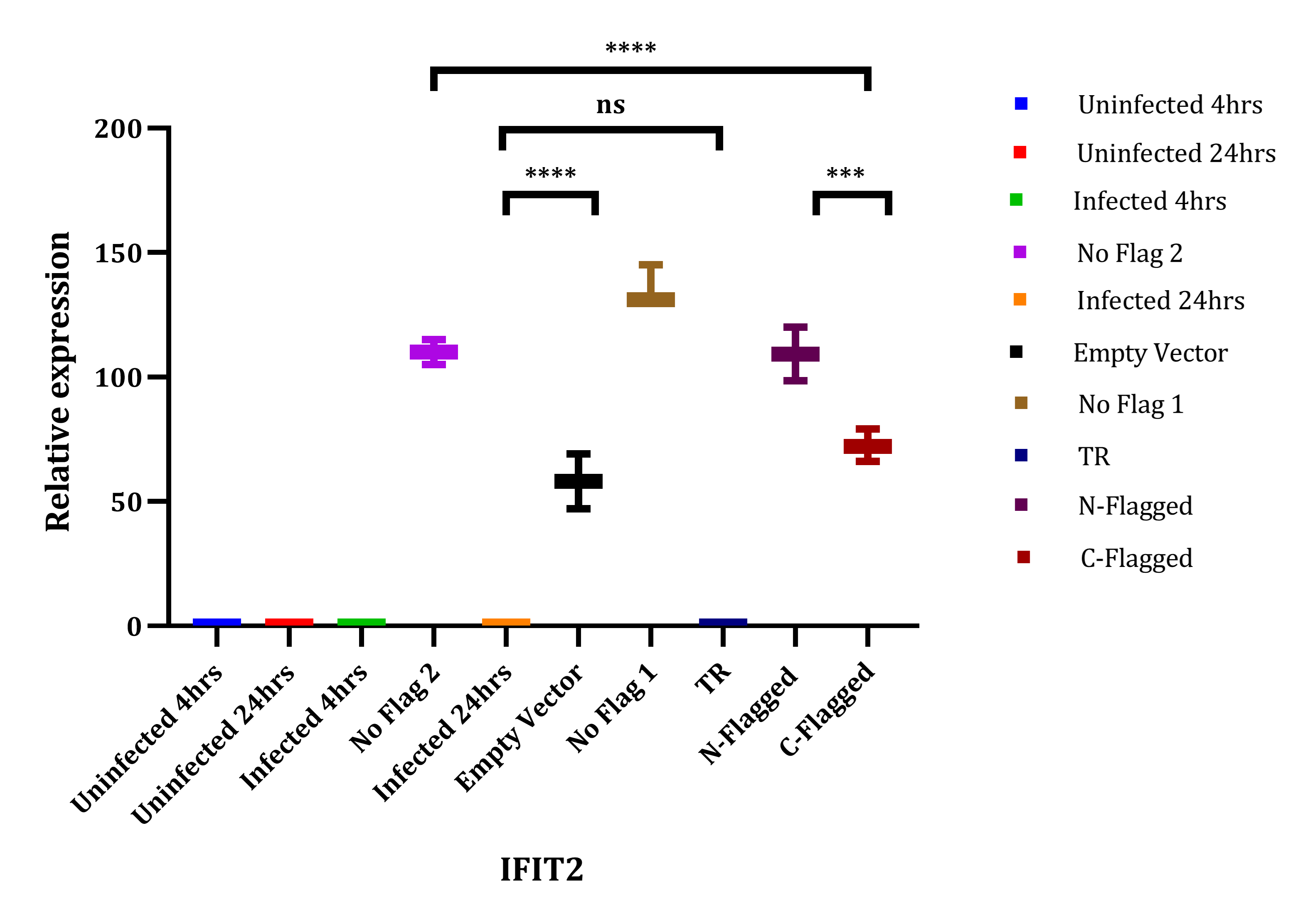

Supplement: Supplementary Figure 1 — Knock-up of IFIT2 using different vector constructs (N-Flagged IFIT2, C-Fagged IFIT2, and No-Flag IFIT2) in M. bovis BCG-infected THP-1 cells assessed by CFUs and relative gene expression. No-flag 1 and No-flag 2 represent technical replicates (A) Intracellular mycobacteria via CFU counts at 24 h post-infection. (B) Relative mRNA expression levels of IFIT2. Included in the graph are the statistical significance (ANOVA and Tukey post-test) levels for the indicated vector treatments against the 24 h infected control. *p-value < 0.05, **p-value <0.01, ***p-value <0.001, ****p-value < 0.0001. TR: Transfection Reagent, ns: not significant. [file DataSheet1.zip › Supplementary Figures/Supplementary Figure 1B.tif]

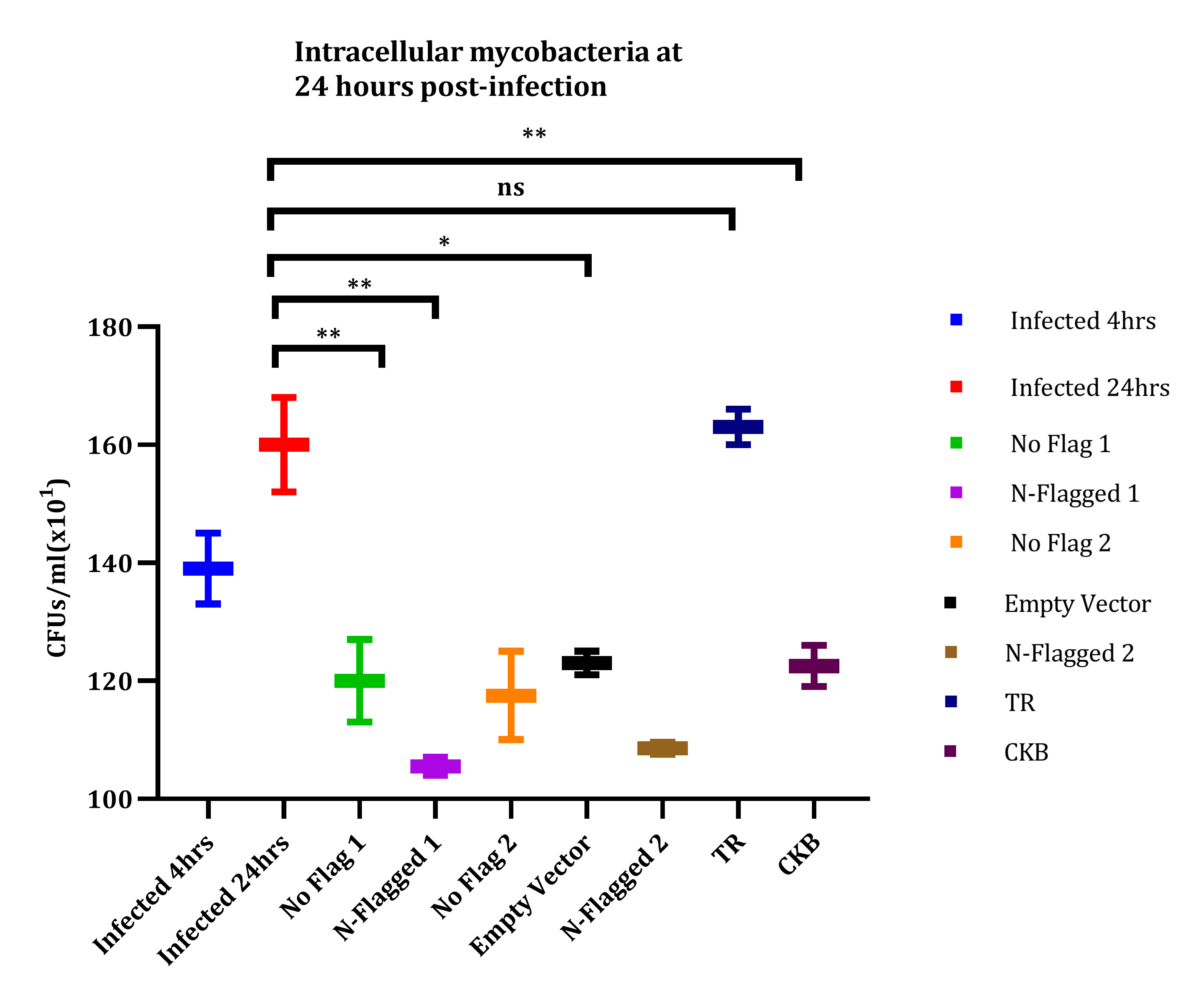

Supplement: Supplementary Figure 1 — Knock-up of IFIT2 using different vector constructs (N-Flagged IFIT2, C-Fagged IFIT2, and No-Flag IFIT2) in M. bovis BCG-infected THP-1 cells assessed by CFUs and relative gene expression. No-flag 1 and No-flag 2 represent technical replicates (A) Intracellular mycobacteria via CFU counts at 24 h post-infection. (B) Relative mRNA expression levels of IFIT2. Included in the graph are the statistical significance (ANOVA and Tukey post-test) levels for the indicated vector treatments against the 24 h infected control. *p-value < 0.05, **p-value <0.01, ***p-value <0.001, ****p-value < 0.0001. TR: Transfection Reagent, ns: not significant. [file DataSheet1.zip › Supplementary Figures/Supplementary Figure 2A.tif]

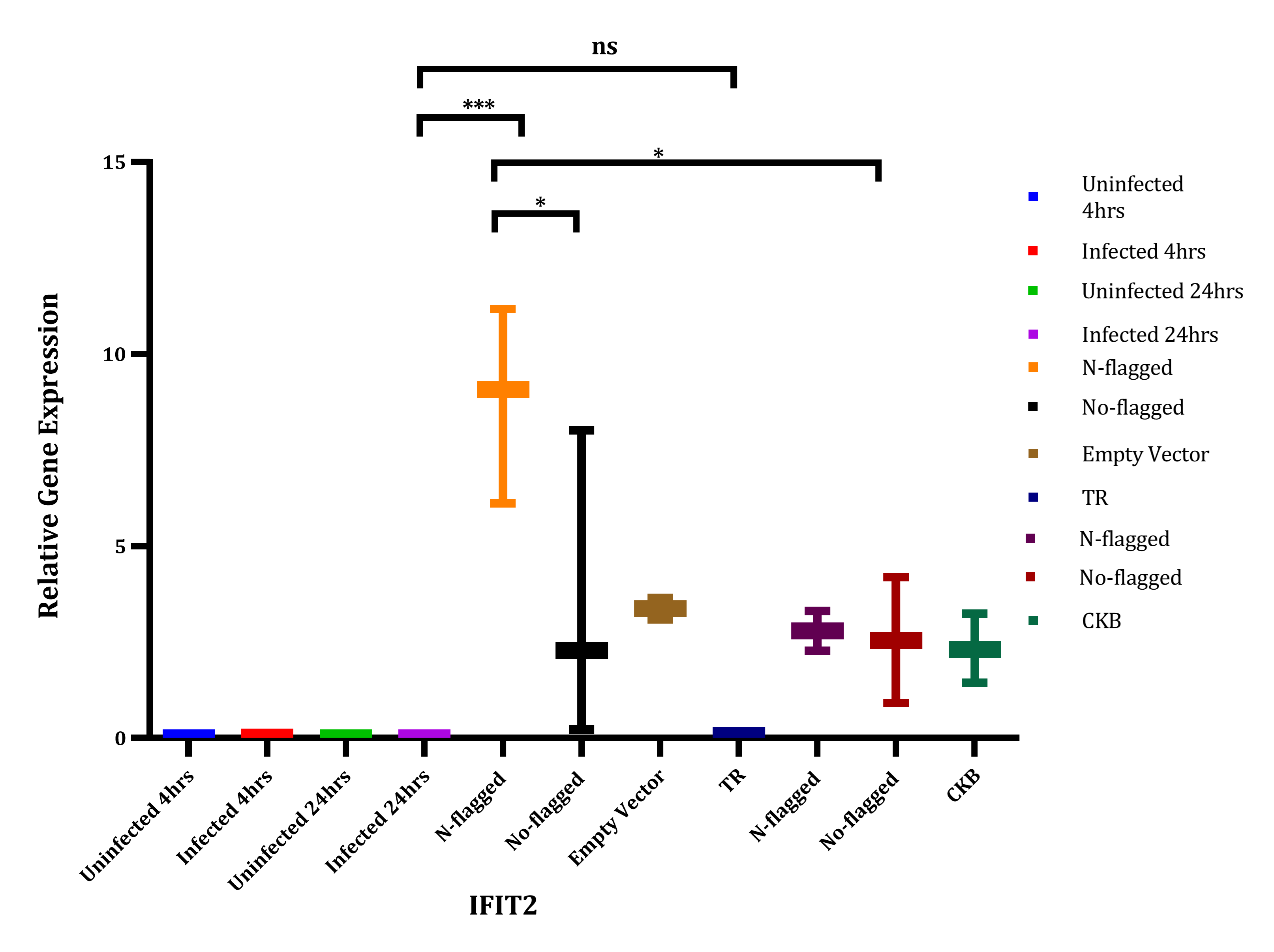

Supplement: Supplementary Figure 1 — Knock-up of IFIT2 using different vector constructs (N-Flagged IFIT2, C-Fagged IFIT2, and No-Flag IFIT2) in M. bovis BCG-infected THP-1 cells assessed by CFUs and relative gene expression. No-flag 1 and No-flag 2 represent technical replicates (A) Intracellular mycobacteria via CFU counts at 24 h post-infection. (B) Relative mRNA expression levels of IFIT2. Included in the graph are the statistical significance (ANOVA and Tukey post-test) levels for the indicated vector treatments against the 24 h infected control. *p-value < 0.05, **p-value <0.01, ***p-value <0.001, ****p-value < 0.0001. TR: Transfection Reagent, ns: not significant. [file DataSheet1.zip › Supplementary Figures/Supplementary Figure 2B.tif]

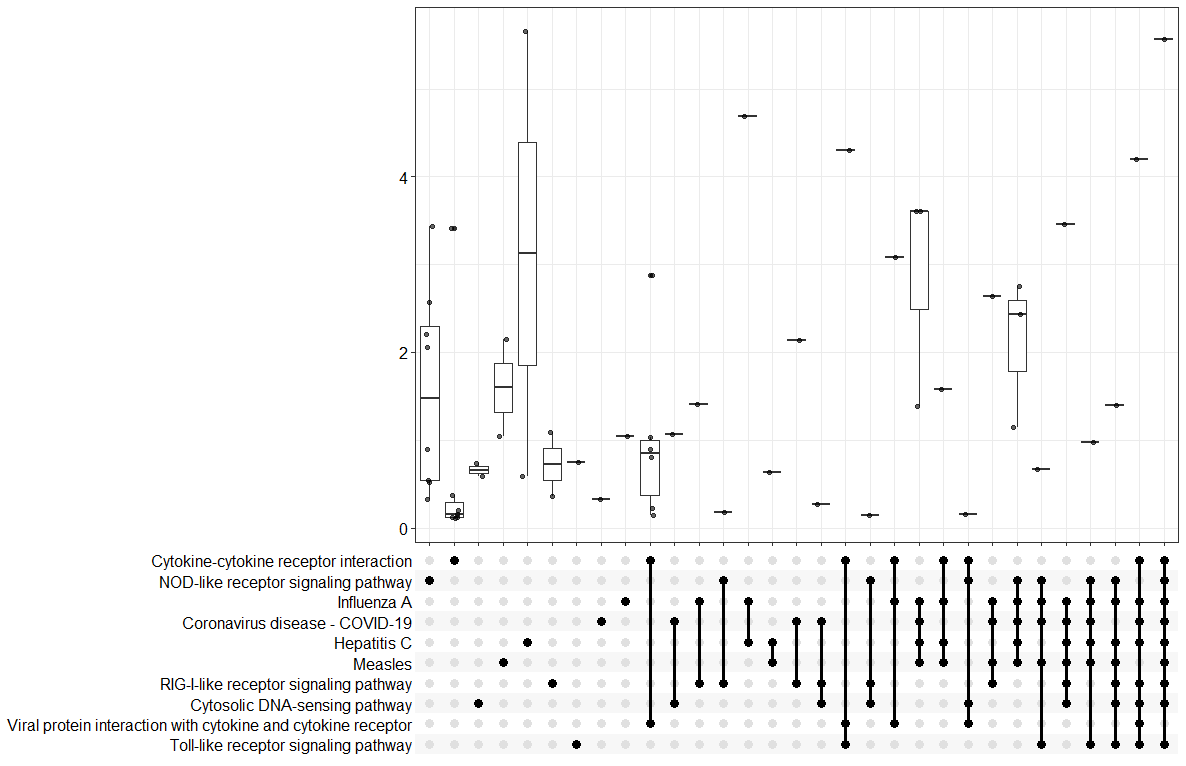

Supplement: Supplementary Figure 1 — Knock-up of IFIT2 using different vector constructs (N-Flagged IFIT2, C-Fagged IFIT2, and No-Flag IFIT2) in M. bovis BCG-infected THP-1 cells assessed by CFUs and relative gene expression. No-flag 1 and No-flag 2 represent technical replicates (A) Intracellular mycobacteria via CFU counts at 24 h post-infection. (B) Relative mRNA expression levels of IFIT2. Included in the graph are the statistical significance (ANOVA and Tukey post-test) levels for the indicated vector treatments against the 24 h infected control. *p-value < 0.05, **p-value <0.01, ***p-value <0.001, ****p-value < 0.0001. TR: Transfection Reagent, ns: not significant. [file DataSheet1.zip › Supplementary Figures/Supplementary Figure 4.tif]

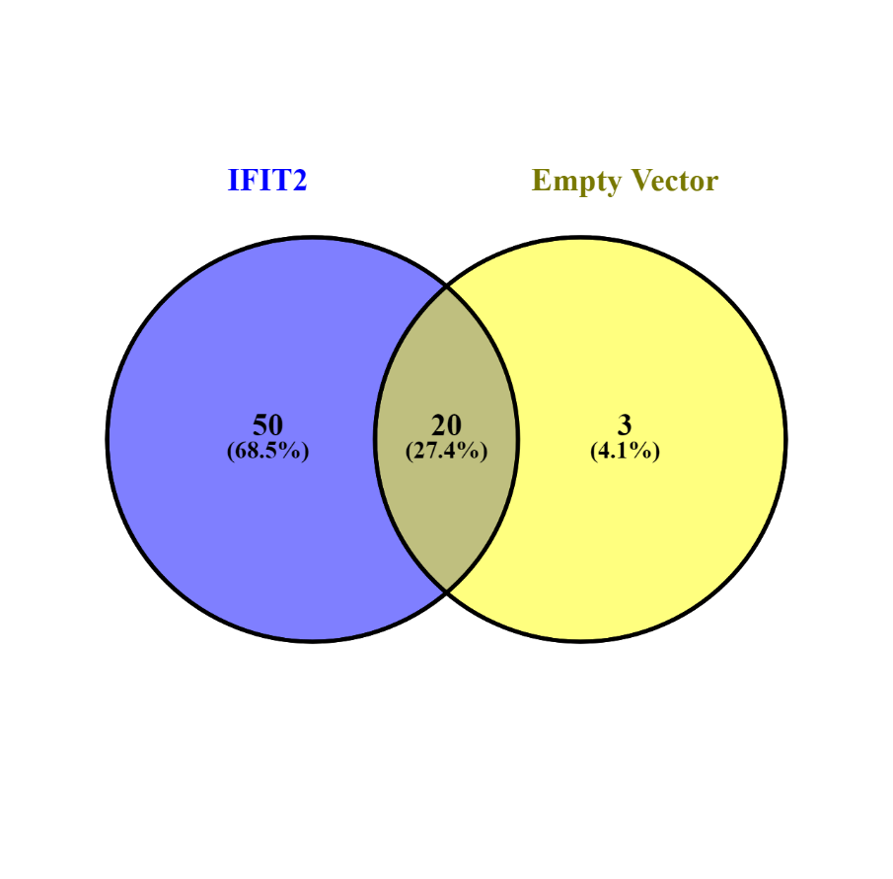

Supplement: Supplementary Figure 1 — Knock-up of IFIT2 using different vector constructs (N-Flagged IFIT2, C-Fagged IFIT2, and No-Flag IFIT2) in M. bovis BCG-infected THP-1 cells assessed by CFUs and relative gene expression. No-flag 1 and No-flag 2 represent technical replicates (A) Intracellular mycobacteria via CFU counts at 24 h post-infection. (B) Relative mRNA expression levels of IFIT2. Included in the graph are the statistical significance (ANOVA and Tukey post-test) levels for the indicated vector treatments against the 24 h infected control. *p-value < 0.05, **p-value <0.01, ***p-value <0.001, ****p-value < 0.0001. TR: Transfection Reagent, ns: not significant. [file DataSheet1.zip › Supplementary Figures/Supplementary Figure 7A.tif]

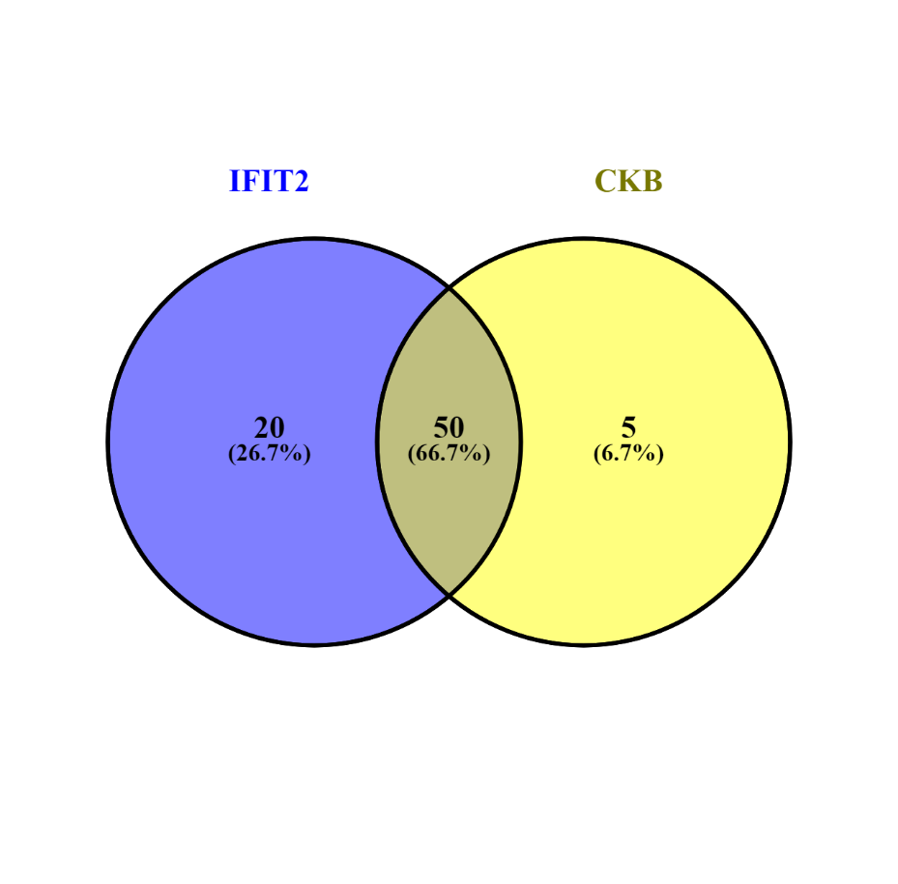

Supplement: Supplementary Figure 1 — Knock-up of IFIT2 using different vector constructs (N-Flagged IFIT2, C-Fagged IFIT2, and No-Flag IFIT2) in M. bovis BCG-infected THP-1 cells assessed by CFUs and relative gene expression. No-flag 1 and No-flag 2 represent technical replicates (A) Intracellular mycobacteria via CFU counts at 24 h post-infection. (B) Relative mRNA expression levels of IFIT2. Included in the graph are the statistical significance (ANOVA and Tukey post-test) levels for the indicated vector treatments against the 24 h infected control. *p-value < 0.05, **p-value <0.01, ***p-value <0.001, ****p-value < 0.0001. TR: Transfection Reagent, ns: not significant. [file DataSheet1.zip › Supplementary Figures/Supplementary Figure 7B.tif]

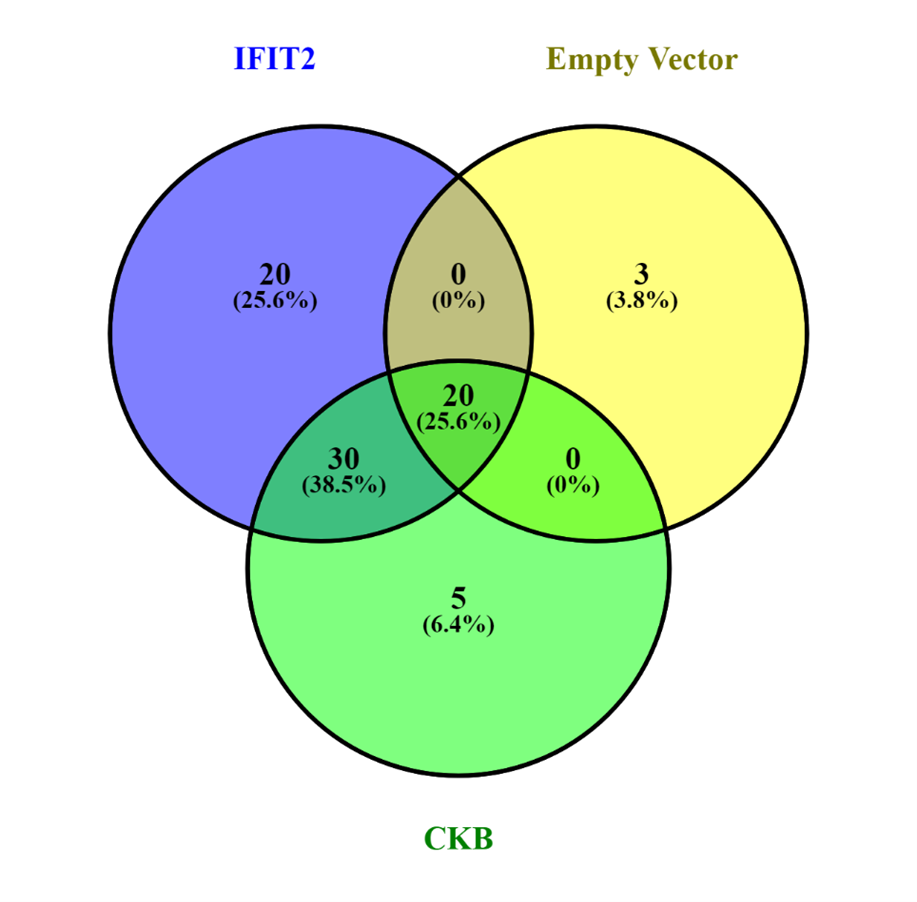

Supplement: Supplementary Figure 1 — Knock-up of IFIT2 using different vector constructs (N-Flagged IFIT2, C-Fagged IFIT2, and No-Flag IFIT2) in M. bovis BCG-infected THP-1 cells assessed by CFUs and relative gene expression. No-flag 1 and No-flag 2 represent technical replicates (A) Intracellular mycobacteria via CFU counts at 24 h post-infection. (B) Relative mRNA expression levels of IFIT2. Included in the graph are the statistical significance (ANOVA and Tukey post-test) levels for the indicated vector treatments against the 24 h infected control. *p-value < 0.05, **p-value <0.01, ***p-value <0.001, ****p-value < 0.0001. TR: Transfection Reagent, ns: not significant. [file DataSheet1.zip › Supplementary Figures/Supplementary Figure 7C.tif]
